# The alkylation of AIM2 by itaconate mediates macrophage PANoptosis during sepsis

**DOI:** 10.1038/s41423-026-01414-x

**Published:** 2026-05-12

**Authors:** Jiamin Ma, Ying Chen, Pei Wang, Yan Zhang, Yi Li, Qing Tu, Xinru Zhao, Xuanqi Yao, Fang Li, Yuan Yuan, Chenwei Wu, Lin Wang, Yuwei Chen, Chenchen Liu, Rui Kang, Daolin Tang, Liangfang Yao, Feng Chen, Jinbao Li

**Affiliations:** 1 Department of Anesthesiology, Shanghai General Hospital, Shanghai Jiao Tong University School of Medicine, Shanghai, China; 2 Department of Anesthesiology and Perioperative Medicine, General Hospital of Ningxia Medical University, Yinchuan, China; 3 Department of Surgery, UT Southwestern Medical Center, Dallas, TX USA; 4 Department of Anesthesiology, Shanghai Ruijin Hospital, Shanghai Jiao Tong University School of Medicine, Shanghai, China

**Keywords:** Itaconate, PANoptosis, AIM2, Macrophages, Sepsis, Infection, Mechanisms of disease

## Abstract

Although the immunometabolite itaconate has long been considered an anti-inflammatory, we found that its profound accumulation paradoxically drives macrophage cell death and pro-inflammatory responses. However, the exact molecular mechanisms underlying itaconate-induced macrophage toxicity remain unclear. Here, we demonstrate that pathophysiologically relevant high concentrations of itaconate covalently alkylate the absent in melanoma 2 (AIM2) protein at the cysteine 113 (C113) residue. Itaconate-mediated C113 alkylation structurally stabilizes the AIM2 protein and triggers a conformational change, enabling it to drive ASC oligomerization, PANoptosome assembly, and subsequent macrophage PANoptosis. Utilizing in vitro lentiviral reconstitution in primary macrophages alongside plasmid-mediated expression in cell lines, we rigorously confirmed that the AIM2 C113A mutation completely abolishes itaconate-induced AIM2 stabilization and PANoptosis. In vivo models further corroborated the pathogenic contribution of this axis to systemic sepsis. Taken together, our findings reveal a novel pro-inflammatory mechanism of itaconate via the post-translational modification of AIM2. The itaconate-AIM2 alkylation axis provides crucial mechanistic insights into macrophage depletion and systemic inflammation, highlighting a potential therapeutic target for severe sepsis.

## Introduction

Sepsis is a life-threatening clinical syndrome characterized by a dysregulated host response to infection, often culminating in systemic hyperinflammation, multi-organ failure, and profound immunosuppression [1–3]. Macrophages lie at the epicenter of this pathogenesis, orchestrating both the initial cytokine storm and the subsequent immunoparalysis [4, 5]. Recent advances in immunometabolism have highlighted that metabolic reprogramming dictates macrophage effector functions [6]. Consequently, identifying key endogenous metabolites that drive pathological transitions in sepsis is crucial for developing targeted therapeutic interventions.

Aconitate decarboxylase 1 (ACOD1) and its catalytic product, itaconate—a tricarboxylic acid (TCA) cycle-derived immunometabolite—have emerged as central regulators of innate immunity. Traditionally, itaconate and its cell-permeable derivatives (e.g., 4-octyl itaconate, 4-OI) are considered anti-inflammatory. These protective effects are predominantly observed in pre-treatment experimental models, where early itaconate intervention mitigates inflammation via Nrf2 activation and the inhibition of glycolysis [7–9]. However, this paradigm is increasingly being challenged. For instance, studies have demonstrated that high concentrations of itaconate promote inflammatory cytokine release and apoptosis in dendritic cells (DCs) [10]. Furthermore, recent evidence has elucidated that in alveolar macrophages (AMs), itaconate exerts unexpected pro-inflammatory effects and exacerbates acute lung injury induced by lipopolysaccharide (LPS) [11]. These findings suggest that the anti-inflammatory properties of itaconate are contingent upon both cell type and concentration. While ACOD1-mediated itaconate synthesis is widely regarded in the literature as an anti-inflammatory mechanism, this notion has been challenged by clinical observations [12]. Transcriptomic analyses of clinical cohorts have revealed that persistently elevated *ACOD1* expression in the peripheral blood of septic patients is paradoxically associated with worse prognosis and increased mortality [13]. This contradiction raises the critical question of whether the sustained accumulation of itaconate during the late or immunosuppressive phases of sepsis triggers previously unrecognized pathological effects, thereby converting its role from immunoprotective to pro-sepsis.

Itaconate is an electrophilic α, β-unsaturated carboxylic acid that can covalently modify cysteine residues on target proteins via Michael addition—a physiological alkylation process termed “itaconation” [14, 15]. This unique chemical property provides a basis for itaconate to function through post-translational modification. Previous studies have reported that the anti-inflammatory properties of itaconate are largely dependent on its alkylating activity (e.g., KEAP1 [14], JAK1 [16], and GPX4 [17]). Furthermore, macrophage programmed cell death mechanisms, particularly the integrated PANoptosis pathway (encompassing pyroptosis, apoptosis, and necroptosis) driven by the PANoptosome, act as terminal amplifiers of inflammation by releasing damage-associated molecular patterns (DAMPs) and executing tissue damage, ultimately leading to multi-organ dysfunction [18, 19]. Whether the metabolic overload of itaconate directly bridges inflammasome activation and PANoptosis via its alkylation effect remains completely unknown.

The aim of this study was to elucidate the molecular mechanism of itaconate in regulating macrophage PANoptosis during sepsis. Here, we demonstrated that both endogenous accumulation and exogenous administration of high-dose itaconate exacerbate systemic hyperinflammation and multi-organ failure. We discovered that during the late immunosuppressive phase, elevated itaconate dynamically induces severe PANoptosis in macrophages through ROS-dependent mitochondrial dysfunction. Mechanistically, we identified that itaconate alkylates the cytosolic DNA-sensor absent in melanoma 2 (AIM2) at the highly conserved Cys113 residue, stabilizing its structure and promoting PANoptosome assembly, which amplifies cytokine storms and tissue injury. Finally, genetic ablation of *Aim2* or point mutation at Cys113 completely rescued macrophages from itaconate-driven PANoptosis and mitigated sepsis severity in vivo. Together, our findings reshape the conceptual framework of itaconate biology, unmasking it as a dual-edged sword that exacerbates sepsis pathogenesis through an AIM2-dependent mechanism, thus offering a novel mechanistic basis for treating high-lethality sepsis.

## Results

### Persistently upregulated ACOD1-itaconate levels are closely associated with poor prognosis in septic patients

**Fig. 1:**
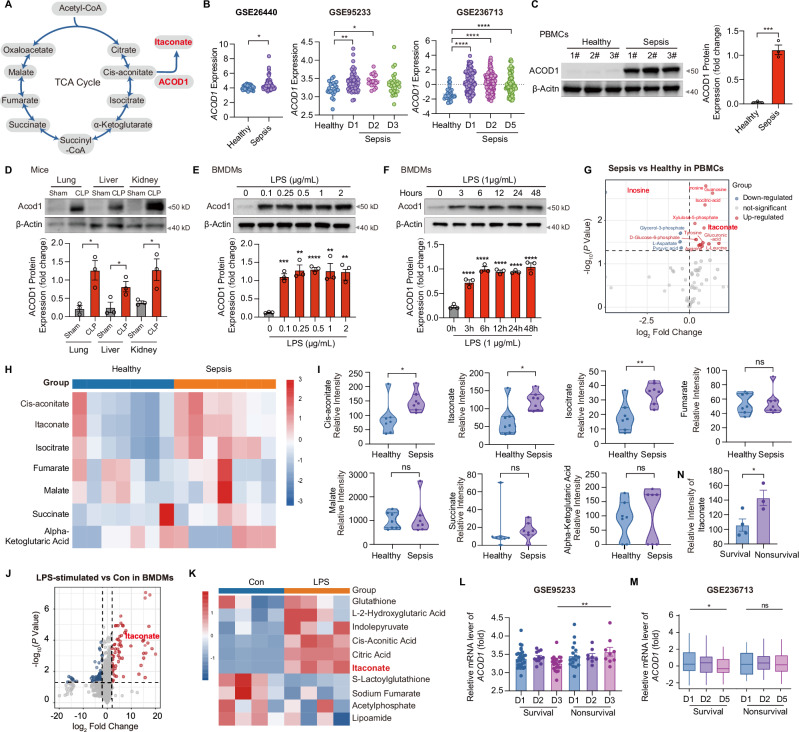
Persistently upregulated ACOD1-itaconate levels are closely associated with poor prognosis in septic patients. **A** Mechanistic diagram of ACOD1- itaconate axis in the TCA cycle. **B** Data from the NCBI GEO database. GSE26440 shows *ACOD1* mRNA levels in whole blood from healthy volunteers (*n* = 32) and pediatric patients with sepsis (*n* = 98) 24 h before admission to the pediatric intensive care unit. GSE95233 shows *ACOD1* mRNA expression in healthy volunteers (*n* = 22) and sepsis patients (*n* = 51) on day 1 (D1), D2 and D3 post-admission. GSE236713 shows *ACOD1* mRNA expression in peripheral blood leukocytes (PBLs) of healthy volunteers (*n* = 30) and sepsis patients (*n* = 164) at D1, D2 and D5 post-ICU admission. ACOD1 protein levels were analyzed by Western blotting in PBMCs from patients (**C**), lung, liver, and kidney tissues from CLP mice (**D**), BMDMs treated with increasing concentrations of LPS for 24 h (**E**), and BMDMs treated with LPS (1 μg/mL) for indicated times (**F**). β-Actin served as a loading control. Data are representative of at least three independent experiments (**C, D, E, F**). Targeted metabolomic analysis of TCA cycle metabolites in PBMCs from healthy volunteers (*n* = 7) and 3-day post-sepsis patients (*n* = 7) via LC-MS,  displayed as a volcano plot (**G**), heatmap (**H**), and statistical analysis of metabolite contents (**I**). Metabolomic profiling of BMDMs in the control (Con, *n* = 4) and LPS-treated (*n* = 4) groups analyzed by LC-MS, visualized using a volcano plot (**J**) and heatmap (**K**). **L** *ACOD1* mRNA expression in whole blood of sepsis survivors (*n* = 34) and non-survivors (*n* = 17) on D1, D2 and D3 post-admission from the GSE95233 dataset. **M** *ACOD1* mRNA expression in whole blood of sepsis survivors (*n* = 118) and non-survivors (*n* = 46) on D1, D2, and D5 post-ICU admission from the GSE236713 dataset. **N** LC-MS quantification of itaconate levels in PBMCs from sepsis survivors (*n* = 4) and non-survivors (*n* = 3). Data are displayed as mean ± SEM. Differences were considered statistically significant at *P < 0.05, **P < 0.01, ***P < 0.001, and ****P < 0.0001

To investigate the role of the ACOD1-itaconate pathway (Fig. 1A) in sepsis, we first analyzed *ACOD1* RNA levels in the peripheral blood of septic patients. We identified three publicly available transcriptomic datasets (GSE26440, GSE95233, and GSE236713) from the NCBI GEO database that included *ACOD1* gene expression profiles (Fig. 1B). Analysis of these datasets revealed a statistically significant elevation in *ACOD1* RNA expression in the peripheral blood of septic patients compared to healthy controls. This increase was observed at multiple time points: day 1 and day 2 after hospital admission, 24 h before intensive care unit (ICU) admission, and days 1, 2, and 5 following ICU admission. Subsequently, we found that the expression of *ACOD1* in peripheral blood mononuclear cells (PBMCs) of patients with sepsis was significantly higher than that of healthy volunteers (Fig. 1C). Furthermore, we observed increased *Acod1* mRNA and ACOD1 protein expression in the lung, liver, and kidney tissues in a polymicrobial sepsis model induced by cecal ligation and puncture (CLP) (Figs. 1D and S1A). Immunofluorescence colocalization revealed that in CLP mouse lung, liver, and kidney tissues, ACOD1 co-localized with M1 macrophages rather than M2 macrophages (Fig. S1B). In primary bone-marrow-derived macrophage cells (BMDMs), LPS concentrations ranging from 0.1 μg/mL to 2 μg/mL induced ACOD1 protein expression. Specifically, LPS at 0.1 μg/mL induced ACOD1 expression comparable to higher concentrations at 24 h, suggesting that ACOD1 expression is saturated at this time point (Fig. 1E). Time-dependent assays further revealed that 1 μg/mL LPS induced ACOD1 protein expression in BMDMs at 6 h (Fig. 1F).

Itaconate, a metabolite of the tricarboxylic acid (TCA) cycle [20], was further examined in metabolomic analyses of TCA-related metabolites in PBMCs from septic patients and healthy controls using liquid chromatography-mass spectrometry (LC-MS) (Figs. S1C and 1G; Supplementary Table 1). Itaconate, cis-aconitate, and isocitrate levels were elevated in septic patients (Fig. 1H, I). In contrast, no significant differences were observed in fumarate, malate, succinate, and alpha-ketoglutarate levels between septic patients and healthy controls, although previous studies have suggested potential alterations in these metabolites. The lack of difference could be attributed to patient heterogeneity. Consistent with these findings, RNA sequencing and metabolomics analysis also showed upregulation of both *Acod1* gene expression and itaconate concentration in LPS-stimulated BMDMs (Figs. 1J, K and S1D–G).

Next, we analyzed public datasets from the NCBI GEO database (GSE95233 and GSE236713) containing clinical survival outcomes. In the GSE95233 dataset, *ACOD1* expression in whole blood was higher at day 3 post-admission in nonsurvival patients compared to survivors (Fig. 1L and Supplementary Table 2). Although this differential expression was not replicated in the GSE236713 dataset, longitudinal analysis revealed a statistically significant decline in *ACOD1* transcript levels in survivors (D5 vs D1: *P* = 0.0158, unpaired t-test), while no change was observed in nonsurviving patients (D5 vs D1: *P* = 0.8318, unpaired t-test) (Fig. 1M and Supplementary Table 3). Additionally, we found that itaconate levels were higher in PBMCs from nonsurvival patients compared to survivors (Fig. 1N). These findings further support that elevated ACOD1-itaconate levels are associated with mortality in sepsis patients.

### The ACOD1-itaconate axis exacerbates systemic inflammation and multi-organ failure in sepsis

**Fig. 2:**
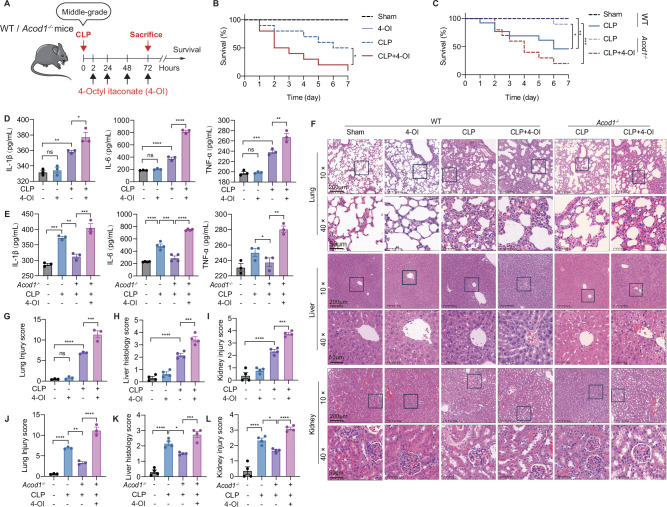
The ACOD1-itaconate axis exacerbates systemic inflammation and multi-organ failure in sepsis. **A** Schematic of the experimental design: WT and *Acod1*^*–/–*^ mice were subjected to CLP followed by intraperitoneal injection of 4-OI (50 mg/kg) at 2, 24, 48, and 72 h post-surgery. Kaplan-Meier survival curves of WT (**B**) and *Acod1*^–^^/–^ mice (**C**) over 7 days following middle-grade sepsis (*n* = 10–15 per group). Serum concentrations of pro-inflammatory cytokines (IL-1β, IL-6, and TNF-α) in WT (**D**) and *Acod1*^–/–^ mice (**E**) at 72 h post-CLP, as measured by ELISA. **F** Representative H&E staining of lung, liver, and kidney tissues. Scale bars, 200 μm for 10× magnification and 50 μm for 40× magnification. Images are representative of three independent experiments. Lung, liver, and kidney injury was scored according to pathological staining in WT (**G**, **H**, **I**) and *Acod1*^*–/–*^ mice (**J**, **K**, **L**). Data are representative of at least three independent experiments (**D**, **E**, **G**, **H**, **I**, **J**, **K**, **L**). Data are displayed as mean ± SEM. Differences were considered statistically significant at *P < 0.05, **P < 0.01, ***P < 0.001, and ****P < 0.0001

4-octyl itaconate (4-OI), a cell-permeable itaconate derivative, is generated via esterification of itaconate with octanol. Compared with other itaconate derivatives, 4-OI retains thiol reactivity comparable to the parent molecule and can be hydrolyzed to release free itaconate intracellularly [21, 22]. To define the functional role of the ACOD1-itaconate axis in polymicrobial sepsis, we subjected wild-type (WT) and global *Acod1*^*–/–*^ mice to CLP-induced experimental sepsis, with or without continuous 4-OI administration to mimic pathological itaconate accumulation during septic conditions (Fig. 2A). Following the CLP challenge, exogenous 4-OI administration drastically curtailed the survival of *Acod1*^*–/–*^ mice and further exacerbated mortality in WT septic mice (Fig. 2B, C). *Acod1*^*–/–*^ mice displayed significantly improved survival relative to WT controls (Fig. 2C), indicating that endogenous ACOD1 activation drives mortality in sepsis. These findings underscore the pro-sepsis role of both endogenous and exogenous itaconate during polymicrobial infection. Consistent with the survival data, 4-OI markedly augmented systemic inflammation, as evidenced by the elevated serum concentrations of cytokines interleukin-1β (IL-1β), IL-6, and tumor necrosis factor-α (TNF-α) (Fig. 2D). While *Acod1* deficiency blunted this systemic inflammatory surge, the protective phenotype was completely neutralized by 4-OI intervention (Fig. 2E). This heightened inflammatory state was accompanied by severe multi-organ dysfunction. Pulmonary analysis revealed that 4-OI exacerbated CLP-induced leukocyte infiltration, interalveolar septal thickening, and alveolar edema, alongside increased lung injury scores and lipid peroxidation (MDA levels) (Figs. 2F, G and S2A). Similarly, 4-OI intensified hepatic damage, characterized by disrupted lobular architecture, hemorrhage, increased liver injury scores, and elevated serum transaminases (AST/ALT) (Figs. 2F, H and S2C). Renal function was also severely compromised, with 4-OI further increasing kidney injury and elevating serum blood urea nitrogen (BUN), creatinine (Cr) and MDA levels beyond the pathological changes observed in the CLP group (Figs. 2F, I and S2E). Collectively, these findings demonstrate that exogenous itaconate drives the progression of systemic hyperinflammation and multi-organ dysfunction, ultimately leading to increased mortality in septic mice. Importantly, the attenuation of sepsis-induced organ injury observed in *Acod1*^*–/–*^ mice was completely reversed by 4-OI supplementation (Figs. 2J–L and S2B, D, F), further confirming the pro-sepsis role of the ACOD1-itaconate axis in this model.

### High-dose itaconate induces macrophage cell death and drives pro-inflammatory responses during the immunosuppressive phase

Given that macrophage cell death plays a central role in regulating the inflammatory response via the release of cytokines and damage-associated molecular patterns (DAMPs), we systematically investigated the effects of various itaconate formulations on macrophage viability. The impact of 4-OI, unmodified itaconate (ITA), and dimethyl itaconate (DMI) at varying concentrations for 24 h was evaluated across RAW264.7 cells, peritoneal macrophages (PMs) and BMDMs (Fig. S3A–C). Cytotoxicity screening revealed distinct, cell-type-specific sensitivity profiles. RAW264.7 cells exhibited compromised viability at concentrations exceeding 250 μM for 4-OI, 500 μM for DMI, and 2.5 mM for ITA (Fig. S3D–F). Conversely, PMs demonstrated greater tolerance, with toxicity thresholds surpassing 500 μM for both 4-OI and DMI, and 10 mM for ITA (Fig. S3G–I). BMDMs displayed an intermediate sensitivity profile ( > 500 μM for 4-OI and DMI; >5 mM for ITA) (Fig. S3J–L). Focusing on BMDMs due to their pathophysiological relevance in sepsis models, SYTOX Green (cell viability dye) viability assays confirmed a concentration-dependent induction of cell death by 1 mM 4-OI, >500 μM DMI, and 10 mM ITA (Fig. S3M–R). Collectively, these data suggest that elevated concentrations of itaconate and its esterified derivatives markedly reduce macrophage viability and induce cell death.

**Fig. 3:**
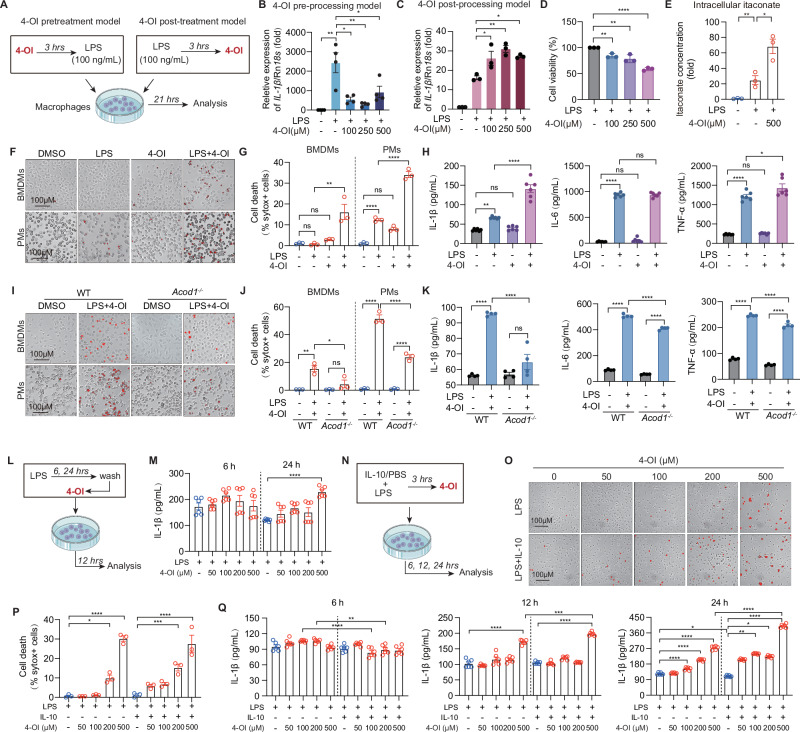
High-dose itaconate induces macrophage cell death and drives pro-inflammatory responses during the immunosuppressive phase. **A** Schematic of the 4-OI pre-treatment and post-treatment experimental setups in macrophages. qRT-PCR analysis of *IL-1β* mRNA expression in BMDMs pre-treated with the indicated concentrations of 4-OI for 3 h prior to LPS stimulation (100 ng/mL) for 21 h (**B**), or pre-treated with LPS (100 ng/mL) for 3 h followed by exposure to 4-OI for 21 h (**C**). **D** Cell viability assessed by CCK-8 assay in BMDMs subjected to the 4-OI post-treatment model. **E** LC-MS quantification of intracellular itaconate in BMDMs stimulated with DMSO, LPS, or LPS + 4-OI for 24 h (n = 3). **F** Cell death evaluation in BMDMs and PMs stimulated with DMSO, LPS, 4-OI, or LPS + 4-OI for 24 h, assessed by SYTOX Green uptake via immunofluorescence. Scale bar, 100 μm. **G** Quantification of the percentage of cells with sytox+ cells among the total cells in BMDMs or PMs. **H** ELISA quantification of IL-1β, IL-6, and TNF-α levels in the supernatants of BMDMs from the post-treatment model. **I** Representative immunofluorescence images of SYTOX Green uptake in WT and *Acod1*^*–/–*^ BMDMs and PMs following LPS + 4-OI stimulation for 24 h. Scale bars, 100 μm. **J** Quantification of the percentage of cells with sytox+ cells among the total cells in WT and *Acod1*^*–/–*^ BMDMs or PMs. **K** ELISA quantification of IL-1β, IL-6, and TNF-α levels in the supernatants of WT and *Acod1*^*–/–*^ BMDMs. **L** Schematic illustrating the in vitro modeling of the immune-active and immunosuppressive phases in macrophages. **M** ELISA quantification of IL-1β secretion in the supernatants of BMDMs across different activation phases. **N** Schematic of high-dose IL-10 treatment used to synthetically mimic the immunosuppressive state in macrophages. **O** Cell death evaluation in BMDMs treated with varying doses of 4-OI in the presence or absence of IL-10, assessed by SYTOX Green immunofluorescence. Scale bars, 100 μm. **P** Quantification of the percentage of cells with sytox+ cells among the total cells in BMDMs. **Q** ELISA quantification of IL-1β secretion from BMDMs treated with varying doses of itaconate in the presence or absence of IL-10. Images are representative of three independent experiments (**F**, **I**, **O**). Data are displayed as mean ± SEM. Differences were considered statistically significant at *P < 0.05, **P < 0.01, ***P < 0.001, and ****P < 0.0001

Previous investigations into the anti-inflammatory effects of itaconate have predominantly relied on 4-OI pre-treatment paradigms [14], whereas its pro-inflammatory properties have largely been characterized using post-treatment models [10]. In bone marrow-derived dendritic cells (BMDCs), 4-OI has been reported to inhibit inflammation at low doses while promoting IL-1β production and inflammatory apoptosis at higher doses [10]. To characterize 4-OI functionality in macrophages, we compared various pre-treatment and post-treatment dosage regimens (Fig. 3A). We observed that the immunomodulatory properties of 4-OI were heavily dependent on the experimental model employed (Fig. 3B, C). CCK-8 assays further demonstrated a progressive, dose-dependent decrease in the viability of LPS-activated BMDMs following 4-OI administration (Fig. 3D). To systematically interrogate the pro-inflammatory mechanisms of high-dose itaconate, we employed the 4-OI post-treatment model for subsequent analyses. Intriguingly, this post-treatment system revealed a sustained production of endogenous intracellular itaconate (Fig. 3E). Using SYTOX Green immunofluorescence, we corroborated that 500 μM 4-OI exacerbated cell death in both LPS-stimulated BMDMs and PMs (Fig. 3F, G). Concomitantly, 4-OI amplified the secretion of IL-1β and TNF-α, but not IL-6, in the supernatants of LPS-activated BMDMs (Fig. 3H), indicating that the sustained production of inflammatory cytokines correlates with a partial loss of cell viability. Importantly, the genetic ablation of *Acod1* significantly attenuated both cell death and the pro-inflammatory response, underscoring its central regulatory role in 4-OI-mediated macrophage activation (Fig. 3I–K).

We sought to elucidate the cellular effects of high-dose itaconate during the transition toward an immunosuppressive microenvironment. To this end, cells were primed with LPS for either 3 h or 24 h to model the immune-active and immunosuppressive phases, respectively, prior to stimulation with escalating doses of itaconate (Fig. 3L). Interestingly, high concentrations of itaconate exerted pro-inflammatory effects exclusively during the late (immunosuppressive) phase, rather than the early (active) inflammatory phase (Fig. 3M). Furthermore, by employing high-dose IL-10 to synthetically recapitulate the immunosuppressive state, we observed that itaconate exhibited a pronounced tendency to exacerbate cell death relative to the IL-10-free controls (Fig. 3N–P). Concurrently, high-dose itaconate robustly triggered the secretion of the pro-inflammatory cytokine IL-1β (Fig. 3Q).

### Itaconate induces PANoptosis in LPS-activated macrophages

**Fig. 4:**
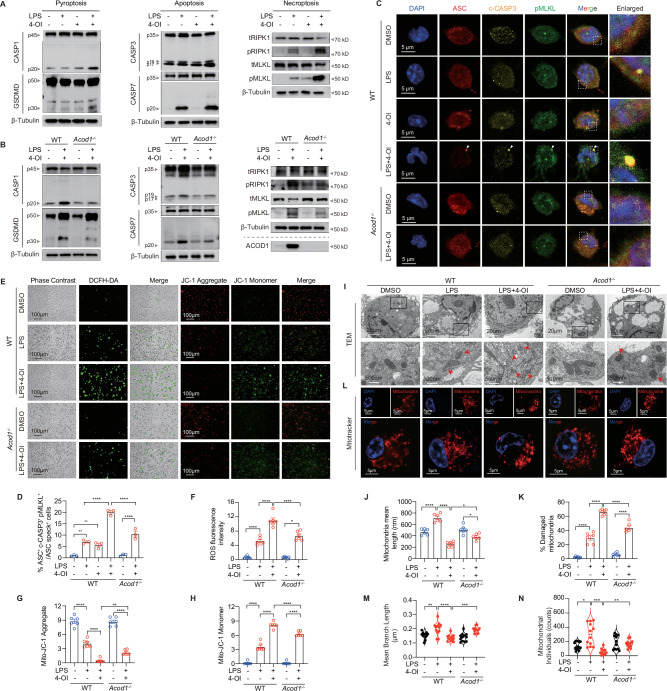
Itaconate induces PANoptosis in LPS-activated macrophages. **A** Immunoblotting analysis of pro- (p45) and activated (p20) caspase-1 (CASP1), pro- (p50) and activated (p30) GSDMD; pro- (p35) and cleaved (p17/19) caspase-3 (CASP3), pro- (p35) and cleaved (p20) caspase-7 (CASP7); and phosphorylated RIPK1 (pRIPK1), total RIPK1 (tRIPK1), phosphorylated MLKL (pMLKL), and total MLKL (tMLKL) in WT BMDMs. **B** Immunoblotting analysis of CASP1, GSDMD, CASP3, CASP7, pRIPK1, tRIPK1, pMLKL, tMLKL, and ACOD1 expression in WT and *Acod1*^*–/–*^ BMDMs treated with the indicated treatments. Data are representative of at least three independent experiments (**A, B**). **C** Representative immunofluorescence images of WT and *Acod1*^*–/–*^ BMDMs at 24 h post-treatment. Arrowheads indicate the ASC speck. Scale bars, 5 μm. **D** Quantification of the percentage of cells containing ASC^+^c-CASP3^+^pMLKL^+^ specks among the ASC speck^+^ cells. **E** Representative images and quantitative analysis of intracellular ROS levels (via DCFH-DA staining; **E,** left) and mitochondrial membrane potential (via JC-1 staining; E, right) Scale bars, 100 μm. **F** Quantification of the mean fluorescent intensity (MFI) of ROS. Quantification of the MFI of JC-1 Aggregate (**G**) and Monomer (**H**). Representative TEM images (**I**), quantification of mean mitochondria branch length (**J**), and the percentage of damaged mitochondria (**K**). Scale bars, 20 μm (top) and 500 nm (bottom). **L** Representative confocal immunofluorescence images of BMDMs pre-treated with LPS for 24 h, stained with MitoTracker Red (mitochondria, red) and Hoechst (nuclei, blue) to visualize mitochondrial morphology. Scale bars, 5 μm. Quantification of mean mitochondria branch length (**M**) and mitochondria counts (**N**). Images are representative of at least three independent experiments (**C**, **E**, **I**, **L**). Data are displayed as mean ± SEM. Differences were considered statistically significant at *P < 0.05, **P < 0.01, ***P < 0.001, and ****P < 0.0001

To elucidate the specific cell death pathways induced by 4-OI in LPS- stimulated BMDMs, we examined the expression and activation of key programmed cell death markers. Compared with the LPS group, the combination of LPS and 4-OI significantly upregulated the activation of pyroptotic (cleaved caspase-1 [CASP1] and gasdermin D [GSDMD]), apoptotic (cleaved caspase-3 [CASP3] and caspase-7 [CASP7]), and necroptotic (phosphorylated receptor-interacting serine/threonine protein kinase 1 [RIPK1] and phosphorylated mixed lineage kinase domain-like pseudokinase [MLKL]) markers (Figs. 4A and S4A). Given the extensive cross-talk between these pathways, these findings suggest the induction of PANoptosis—a multifaceted programmed cell death mechanism—by 4-OI [23, 24]. Consistent with this, *Acod1* knockout markedly attenuated the levels of PANoptosis-associated proteins, underscoring the role of the endogenous ACOD1-itaconate axis in this process (Figs. 4B and S4B). Immunofluorescence imaging revealed that while PYCARD/ASC, cleaved caspase-3 (c-CASP3), and p-MLKL were diffusely distributed in control or LPS-treated cells, 4-OI treatment in LPS-primed BMDMs induced the formation of large ASC specks that highly colocalized with p-MLKL and c-CASP3 puncta (Fig. 4C, D). PYCARD/ASC specks are supramolecular complexes central to inflammasome activation, which drive inflammatory cell death pathways. Notably, the number of these specks was significantly reduced following *Acod1* knockout (Fig. 4C, D). Overall, these findings indicate that 4-OI induces PANoptosis in LPS-stimulated macrophages.

Considering the pivotal role of mitochondria in orchestrating cell death and inflammatory signaling, we next investigated whether mitochondrial dysfunction underpins ACOD1/itaconate-mediated PANoptosis. 4-OI treatment drastically elevated reactive oxygen species (ROS) production and dissipated the mitochondrial membrane potential, as evidenced by a shift from JC-1 aggregates to monomers (Fig. 4E–H). Conversely, these effects were largely rescued by *Acod1* deficiency. Transmission electron microscopy (TEM) further confirmed that 4-OI induced severe ultrastructural damage, including mitochondrial swelling and cristae fragmentation (Fig. 4I–K). Analysis of mitochondrial dynamics revealed that while LPS alone appeared to trigger a compensatory protective response—manifested by increased mitochondrial fusion with longer average branch lengths and higher counts—4-OI treatment collapsed this homeostatic balance, resulting in fragmented and diminished mitochondrial networks (Fig. 4L–N). Concomitantly, 4-OI shifted the balance of mitochondrial BCL-2 family proteins by escalating pro-apoptotic BAX and suppressing anti-apoptotic BCL-2 expression, a phenotype that was reversed upon *Acod1* knockout (Fig. S4C, D). Collectively, these data suggest that high-dose itaconate drives ROS-dependent mitochondrial dysfunction and homeostatic collapse, ultimately leading to PANoptosis in LPS-stimulated macrophages.

### Itaconate drives macrophage PANoptosis and exacerbates sepsis via AIM2-dependent PANoptosome assembly

**Fig. 5:**
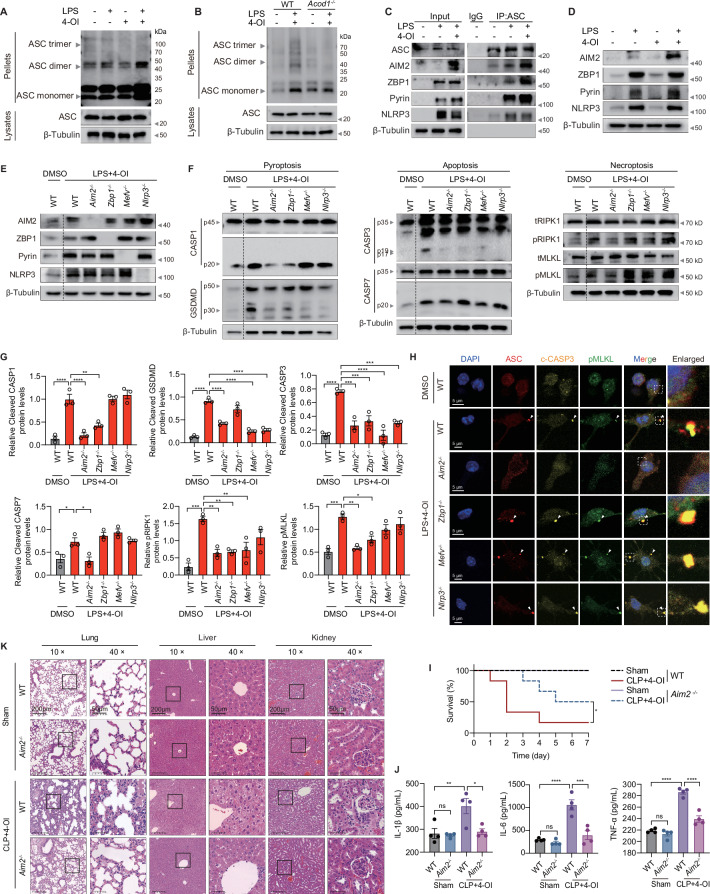
Itaconate drives macrophage PANoptosis and exacerbates sepsis via AIM2-dependent PANoptosome assembly. Immunoblotting analysis for ASC oligomerisation in WT (**A**) and *Acod1*^*–/–*^ BMDMs (**B**). **C** Co-immunoprecipitation analysis of PANoptosome assembly and immunoblotting analysis of ASC, AIM2, ZBP1, Pyrin, and NLRP3 in LPS-stimulated BMDMs treated with or without 4-OI. **D** Immunoblotting analysis of AIM2, ZBP1, Pyrin, and NLRP3 protein levels in BMDMs. Immunoblotting analysis of AIM2, ZBP1, Pyrin, and NLRP3 expression (**E**), as well as CASP1, GSDMD, CASP3, CASP7, pRIPK1, tRIPK1, pMLKL, and tMLKL expression (**F**) in WT, *Aim2*^*–/–*^, *Zbp1*^*–/–*^, *Mefv*^*–/–*^, and *Nlrp3*^*–/–*^ BMDMs. Data are representative of at least three independent experiments. **G** Quantification statistics of cleaved-CASP1, cleaved-GSDMD, cleaved-CASP3, cleaved-CASP7, pRIPK1, and pMLKL expression from the indicated BMDM genotypes. **H** Representative immunofluorescence images of WT, *Aim2*^*–/–*^, *Zbp1*^*–/–*^, *Mefv*^*–/–*^, and *Nlrp3*^*–/–*^ BMDMs at 24 h post-treatment with LPS and 4-OI. Arrowheads indicate ASC speck. Scale bars, 5 μm. **I** Kaplan-Meier survival curves of WT and *Aim2*^*–/–*^ mice subjected to middle-grade sepsis (CLP) followed by intraperitoneal injected of 4-OI (50 mg/kg) at 2, 24, 48, and 72 h post-surgery. **J** ELISA quantification of plasma biomarkers (IL-1β, IL-6, and TNF-α). **K** Representative H&E staining of lung, liver, and kidney tissues (Scale bars, 200 μm for 10× magnification and 50 μm for 40× magnification). Images are representative of three independent experiments (**H, K**). Data are displayed as mean ± SEM. Differences were considered statistically significant at *P < 0.05, **P < 0.01, ***P < 0.001, and ****P < 0.0001

The PANoptosome is a molecular scaffold comprising sensors such as AIM2, Z-DNA binding protein 1 (ZBP1), NLRP3, and Pyrin (encoded by *Mefv*), alongside the adaptor protein PYCARD/ASC and downstream effectors including GSDMD, CASP3/7, and MLKL [25]. Upon inflammasome activation, ASC recruits the cysteine protease CASP1, facilitating its proteolytic cleavage and activation [26]. Furthermore, ASC directly interacts with PANoptosome components (e.g., ZBP1 and RIPK3), bridging inflammasome signaling with other death pathways [27]. ASC oligomerization is essential in this activation process [28]. To elucidate the molecular underpinnings of 4-OI-triggered inflammasome activation, we first examined ASC oligomerization dynamics in BMDMs. Comparative analysis revealed that combined LPS and 4-OI treatment markedly augmented ASC dimerization relative to LPS mono-stimulation, an effect that was substantially attenuated upon *Acod1* deficiency (Figs. 5A, B and S5A, B). Subsequent co-immunoprecipitation (Co-IP) assays were performed to identify the specific sensors associated with this process. While basal interactions between ASC and the sensors AIM2, ZBP1, and Pyrin were detectable in LPS-primed BMDMs, these interactions were significantly potentiated by 4-OI co-treatment (Fig. 5C). Western blot analysis confirmed that this 4-OI-mediated enhancement of ASC-sensor recruitment correlated with a significant upregulation of AIM2 protein levels, whereas the expression of ZBP1, Pyrin, and NLRP3 remained unchanged (Figs. 5D and S5C). Interestingly, RT-PCR analysis showed no significant differences in the mRNA levels of *Aim2*, *Zbp1*, *Mefv*, or *Nlrp3*, suggesting that 4-OI regulates AIM2 primarily at the protein level (Fig. S5D).

We subsequently investigated the roles of specific sensors—AIM2, ZBP1, Pyrin, and NLRP3—in mediating 4-OI-induced PANoptosis following LPS stimulation. Intriguingly, *Aim2* deficiency, but not the deficiency of *Zbp1*, *Mefv*, or *Nlrp3*, markedly blunted the activation of PANoptosis-associated molecules, and significantly reduced cell death and IL-1β secretion (but not IL-6 or TNF-α) (Figs. 5E–G and S5E–G). Immunofluorescence analysis further corroborated that the formation of p-MLKL/c-CASP3/ASC-positive PANoptosome puncta was significantly diminished in *Aim2*^*–/–*^ cells compared to WT controls (Figs. 5H and S5H). Consistent with these cellular findings, ELISA indicated that 4-OI treatment promoted the release of AIM2 into the culture supernatants of LPS-stimulated BMDMs (Fig. S5I). Furthermore, immunofluorescence staining of tissues demonstrated elevated AIM2 expression in the CLP + 4-OI group compared to the CLP-only group (Fig. S5J, K). Proximity ligation assays (PLA) further quantified the enhanced molecular proximity between ASC and AIM2 in 4-OI-treated cells (Fig. S6A). Finally, confocal microscopy confirmed that AIM2 was highly colocalized with ASC specks in BMDMs challenged with LPS and 4-OI (Fig. S6B, C). Collectively, these findings demonstrate that 4-OI specifically upregulates AIM2 protein expression and that *Aim2* deficiency effectively rescues BMDMs from LPS + 4-OI-induced PANoptosis.

To further validate the pathological significance of the AIM2 in sepsis-associated organ injury in vivo, we utilized *Aim2*-deficient mice in the 4-OI-challenged CLP model. Significantly, *Aim2*^*–/–*^ mice conferred a substantial survival advantage compared with their WT (Fig. 5I). Multiplex cytokine analysis revealed that global *Aim2* deficiency significantly attenuated systemic inflammation, as evidenced by markedly reduced serum concentrations of IL-1β, IL-6, and TNF-α following CLP surgery and 4-OI administration (Fig. 5J). Histopathological and biochemical analyses revealed that *Aim2*^*–/–*^ mice exhibited markedly attenuated multi-organ damage in the lungs, liver, and kidneys, with significantly reduced lipid peroxidation (Figs. 5K and S6D–F). Taken together, these data strongly suggest that *Aim2* ablation attenuates itaconate-exacerbated systemic inflammation and mitigates multi-organ dysfunction in septic mice.

### Itaconate enhances AIM2 stability via alkylation at Cys113

**Fig. 6:**
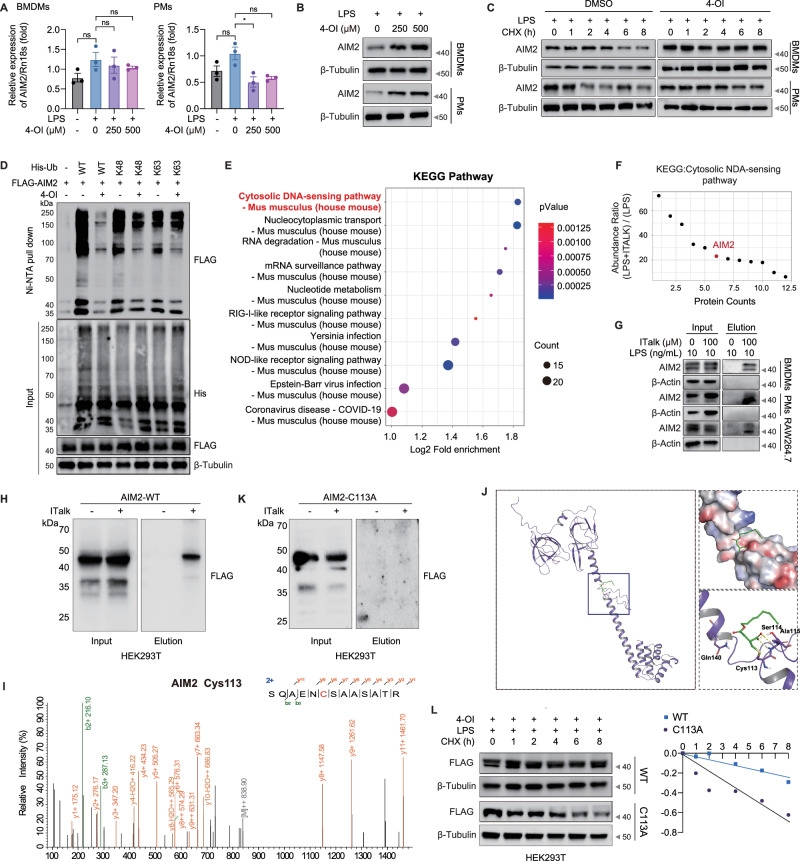
Itaconate enhances AIM2 stability via alkylation at Cys113. qRT-PCR analysis of* Aim2* RNA (**A**) and immunoblotting analysis of AIM2 protein (**B**) levels in LPS-activated BMDMs and PMs treated with the indicated concentration of 4-OI for 24 h. **C** Protein half-life evaluation (CHX chase assay) of AIM2 in BMDMs and PMs pretreated with LPS and 4-OI (500 μM) for 24 h prior to cycloheximide (CHX, 100 μg/mL) administration at the specified time points. **D** Immunoblotting analysis of whole-cell lysates and nickel-nitrilotriacetic acid (Ni-NTA) pull-down fractions from HEK293T cells transfected with FLAG-AIM2 and K-only ubiquitin mutants. At 24 h post-transfection, cells were treated with 4-OI (500 μM) for 24 h. **E** KEGG pathway enrichment analysis of the identified ITalk-labeled target proteins. The x-axis represents Log2 (Fold enrichment), circle size indicates the number of target proteins in the specific pathway, and color gradients reflect statistical significance (*P*-value). **F** Identified target proteins within the cytosolic DNA-sensing pathway, prominently featuring AIM2. **G** Validation of direct ITalk labeling of endogenous AIM2 in BMDMs, PMs and RAW264.7 cells. **H** HEK293T cells were transfected with AIM2-FLAG overexpression plasmid for 24 h and then treated with 100 μM ITalk or an equivalent volume of DMSO for 12 h. The samples before and after enrichment were analyzed by anti-FLAG immunoblotting. **I** High-resolution LC-MS/MS spectra identifying Cys113 as the specific site of ITalk alkylation on mouse AIM2. **J** 3D molecular model visualizing the covalent docking of ITalk onto Cys113 within the mouse AIM2 structural domain. **K** HEK293T cells were transfected with a mouse C113A-mutated AIM2-FLAG overexpression plasmid for 24 h and then treated with 100 μM ITalk or an equivalent volume of DMSO for 12 h. The samples before and after enrichment were analyzed by anti-FLAG immunoblotting. **L** CHX chase assay evaluating the half-life of WT and C113A-mutated AIM2 in HEK293T cells following pre-treatment with LPS and 4-OI. Data are displayed as mean ± SEM. Differences were considered statistically significant at *P < 0.05, **P < 0.01, ***P < 0.001, and ****P < 0.0001

To elucidate the mechanisms underlying 4-OI-mediated AIM2 upregulation, we treated BMDMs and PMs with escalating concentrations of 4-OI. This treatment induced a dose-dependent accumulation of AIM2 protein, whereas *Aim2* mRNA levels remained largely unperturbed (Fig. 6A, B). Subsequent half-life evaluation demonstrated that 4-OI significantly enhanced AIM2 protein stability (Fig. 6C), indicating post-translational regulation rather than transcriptional activation. Mechanistic dissection of AIM2 clearance revealed a multifactorial degradation process involving both the ubiquitin-proteasome system (UPS) and the autophagy-lysosomal pathway (Fig.S7A). To delineate the ubiquitination dynamics, we performed cell-based ubiquitination assays using lysine-restricted ubiquitin mutants. Strikingly, 4-OI treatment markedly abrogated both K48- and K63-linked polyubiquitination of AIM2 (Fig. 6D).

Itaconate and its derivatives harbor electrophilic α, β-unsaturated carboxylic moieties, enabling covalent modification of protein cysteine residues via Michael addition—a process termed “itaconation” [29]. Given that such alkylation can profoundly alter protein function and stability, we hypothesized that 4-OI stabilizes AIM2 via direct cysteine modification. To investigate this, we employed the bioorthogonal probe itaconate-alkyne (ITalk) [29] to map itaconate-induced alkylation events in macrophages utilizing click chemistry (Fig.S7B). Following ITalk labeling in RAW264.7 cells, chemoproteomic profiling identified 3,346 significantly enriched proteins (log2 abundance ratio > 1), successfully capturing known endogenous targets of itaconation, including NLRP3 [21], Kelch-like ECH-associated protein 1 (KEAP1) [14], lactate dehydrogenase A (LDHA) [14], and JAK1 [16] (Fig.S7C–E). Furthermore, we mapped 792 proteins containing 989 specific modification sites. Kyoto Encyclopedia of Genes and Genomes (KEGG) pathway analysis revealed an enrichment of these targets in cytosolic DNA-sensing pathways, prominently featuring AIM2 (Figs. 6E, F and S7D). Correspondingly, direct ITalk labeling of endogenous mouse AIM2 was confirmed in primary BMDMs, PMs, and RAW264.7 cells (Fig. 6G).

To definitively validate the direct interaction of AIM2, we overexpressed mouse AIM2 in HEK293T cells and exposed them to the ITalk probe. Subsequent immunoprecipitation of AIM2 revealed robust ITalk incorporation (Fig. 6H). High-resolution LC-MS/MS analysis pinpointed Cys113 as the critical cysteine residue undergoing alkylation (Fig. 6I). Molecular modeling further visualized the covalent docking of ITalk onto Cys113 within the mouse AIM2 structural domain (Fig. 6J). To verify the functional significance of this modification, we transfected a C113A-mutated AIM2 plasmid into 293T cells, and found that ITalk labeling of AIM2 was absent (Fig. 6K). Furthermore, the effect of 4-OI on extending the half-life of AIM2 was abrogated by the Cys113 mutation (Fig. 6L). To screen for E3 ubiquitin ligases downstream of AIM2, we overexpressed WT AIM2 and the C113A mutant in HEK293T cells and performed Co-IP coupled with mass spectrometry. This analysis yielded 70 common interacting proteins from both groups, among which only one E3 ubiquitin ligase, WW domain containing E3 ubiquitin protein ligase 1 (WWP1), was identified (Fig.S7F). Notably, WB validation demonstrated a significantly increased interaction between AIM2 and WWP1 upon C113A mutation compared to the WT (Fig.S7G). Collectively, these results suggest that 4-OI directly alkylates AIM2 at Cys113, thereby preventing its polyubiquitination and promoting its post-translational stabilization.

### Mutation of AIM2 at Cys113 alleviates itaconate-induced PANoptosome assembly and PANoptosis

**Fig. 7:**
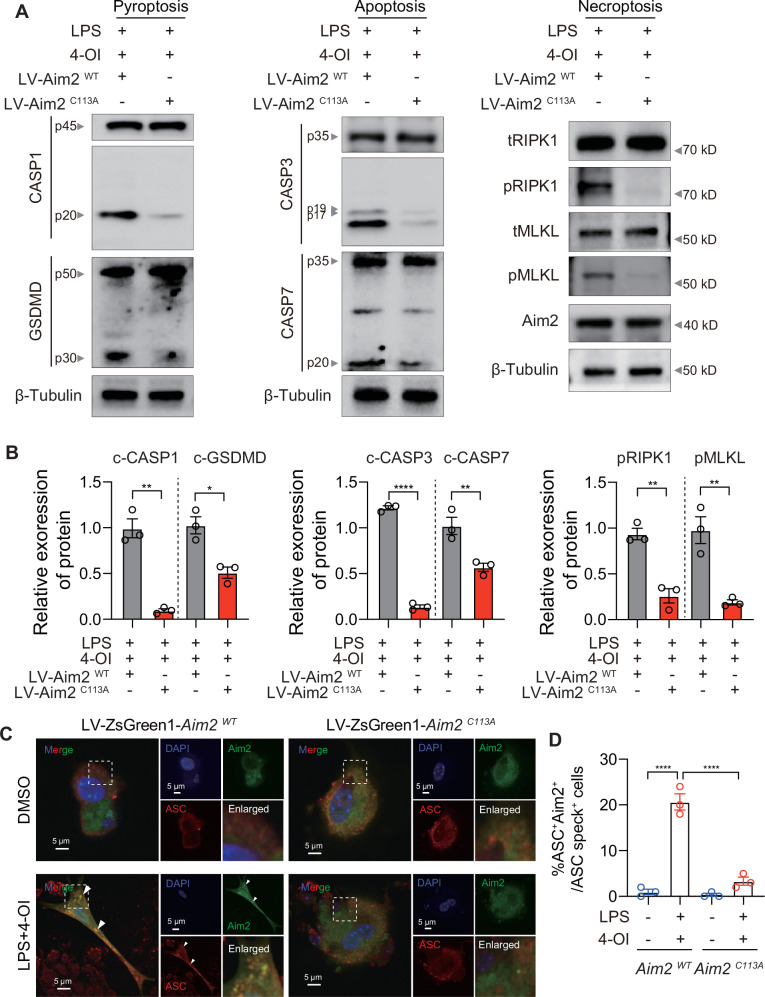
Mutation of AIM2 at Cys113 alleviates itaconate-induced PANoptosome assembly and PANoptosis. Immunoblotting analysis (**A**) and corresponding quantification (**B**) of CASP1, GSDMD, CASP3, CASP7, pRIPK1, tRIPK1, pMLKL, and tMLKL expression in *Aim2*^–/–^ BMDMs reconstituted with LV-Aim2 WT or LV-Aim2 C113A. **C** Representative immunofluorescence images of *Aim2*^–/–^ BMDMs reconstituted with LV-EsGreen1-Aim2 WT or LV- EsGreen1-Aim2 C113A following LPS and 4-OI treatment. Arrowheads indicate the ASC speck. Scale bar, 5 μm. Images are representative of three independent experiments. **D** Quantification of the percentage of cells with AIM2^+^ specks among the ASC speck^+^ cells. Data are displayed as mean ± SEM. Differences were considered statistically significant at *P < 0.05, **P < 0.01, ***P < 0.001, and ****P < 0.0001

To strictly validate whether the specific alkylation of AIM2 at Cys113 determines cellular susceptibility to itaconate-induced PANoptosis, we performed genetic reconstitution experiments in *Aim2*^–/–^ BMDMs. We engineered lentiviral vectors to stably express either the EsGreen1-tagged wild-type AIM2 (EsGreen1-AIM2 WT) or the alkylation-deficient mutant (EsGreen1-AIM2 C113A). At a multiplicity of infection (MOI) of 100, the viral transduction efficiency reached the experimental requirement, ensuring stable expression of the AIM2 WT and C113A mutant in target cells (Fig.S8A). Next, we assessed the activation profiles of the PANoptosis execution cascade via Western blot analysis. As expected, 4-OI treatment combined with LPS robustly triggered the cleavage and activation of key pyroptotic, apoptotic, and necroptotic effector proteins in *Aim2*^–/–^ BMDMs reconstituted with AIM2 WT. In stark contrast, cells expressing the AIM2 C113A mutant exhibited markedly reduced levels of all these death-related proteins (Fig. 7A, B). To further characterize the impact of the Cys113 mutation on the supramolecular machinery of cell death, we performed confocal immunofluorescence imaging to visualize the assembly of the PANoptosome scaffold. Upon LPS and 4-OI stimulation, cells expressing AIM2 WT displayed striking intracellular colocalization of AIM2 and ASC, forming massive characteristic specks. Conversely, the formation of AIM2-ASC colocalized specks was markedly decreased in AIM2 C113A-expressing BMDMs, confirming that the loss of Cys113 alkylation drastically impaired the physical recruitment and scaffolding of ASC (Fig. 7C, D). Collectively, these data provide compelling phenotypic and biochemical evidence that the specific covalent modification of AIM2 at Cys113 by 4-OI is a prerequisite for PANoptosome assembly and the execution of extensive macrophage PANoptosis.

## Discussion

Sepsis is characterized by a profoundly dysregulated host response to infection, biphasically manifesting as an initial hyperinflammatory cytokine storm followed by severe immunosuppression [30]. Recent advances in immunometabolism have highlighted metabolic reprogramming as a central determinant of macrophage effector functions, wherein the ACOD1-itaconate axis has traditionally been celebrated as a major anti-inflammatory brake [14, 31]. However, by integrating clinical transcriptomics, multi-omics profiling, and advanced biochemical analyses, our study uncovers a largely unrecognized pro-inflammatory role of itaconate biology in sepsis. We demonstrate that during the late, immunosuppressive phase, persistent accumulation of high-dose itaconate loses its protective properties and instead acts as a primary driver of macrophage PANoptosis by directly alkylating and stabilizing the cytosolic DNA sensor AIM2 (Fig. S9). This discovery fundamentally reshapes our conceptual framework of itaconate, defining it as a pathophysiological “double-edged sword” that ultimately exacerbates systemic hyperinflammation and multi-organ failure in late-stage sepsis.

First, our findings reconcile a long-standing contradiction between experimental models and clinical realities. Previous paradigms, heavily reliant on prophylactic or early-intervention models, posited that itaconate and its cell-permeable derivative, 4-OI, confer anti-inflammatory protection primarily via NRF2 activation or glycolysis inhibition. In stark contrast, our longitudinal analysis of independent clinical cohorts (GSE95233 and GSE236713) and metabolomic profiling firmly establish that persistently elevated *ACOD1* transcription and endogenous itaconate accumulation in peripheral blood are highly predictive of poor prognosis and increased mortality in septic patients. Using in vitro models, we demonstrated that sustained itaconate stimulation directly promotes multi-organ dysfunction associated with sepsis. Notably, even genetic ablation of *Acod1* did not abolish this damaging effect, underscoring that the pathological accumulation of itaconate, rather than its synthesis machinery, plays a decisive role in exacerbating sepsis pathology. By utilizing varied administration regimens in vitro, we confirmed that the immunomodulatory nature of itaconate is strictly temporally regulated and dose-dependent. While early intervention might offer buffering effects, exposure to high-dose itaconate during the late immunosuppressive phase (modeled by extensive LPS priming or IL-10 presence) dramatically shifts its role toward inducing pro-inflammatory cell death and IL-1β release. Consistent with our findings, a recent study reported that BRD3 drives the expression of ACOD1 and the production of itaconate and multiple pro-inflammatory cytokines in macrophages via the TRIM21-mediated CREBBP-CREB1 signaling axis, thereby amplifying inflammation and promoting tissue injury [32]. This cautions against the indiscriminate therapeutic use of ACOD1 agonists or itaconate supplementation, which could independently precipitate disastrous outcomes if administered outside a narrow early therapeutic window.

Previous metabolic profiling has indicated that the intracellular concentration of itaconate can accumulate to levels as high as 2–8 mM in the macrophages [33]. To pathophysiologically recapitulate this robust itaconate accumulation in vitro, we selected a working concentration of 500 μM for 4-OI in our subsequent functional assays. Notably, the utilization of this specific concentration is well-precedented in prior literature demonstrating the pro-inflammatory and cytotoxic effects of itaconate derivatives [10]. Our results reveal that itaconate, in conjunction with LPS stimulation, triggers macrophage PANoptosis. This form of programmed cell death is orchestrated by the PANoptosome—a multimeric complex assembled upon ASC oligomerization that serves as a scaffold for the sequential engagement of inflammasomes and downstream effector proteins. In the context of unresolved infection, rampant macrophage death not only dismantles innate immune defenses but also acts as the terminal amplifier of inflammation via the massive release of damage-associated molecular patterns (DAMPs) [34]. This massive macrophage death is an important cause of multiple organ dysfunction caused by itaconate in mice. Furthermore, our data elucidate that high doses of itaconate potently trigger reactive oxygen species (ROS) production and reduce mitochondrial repair capacity in sepsis, causing mitochondrial dysfunction. Notably, recent literature has reported that itaconate—whether administered exogenously or derived endogenously, both in vivo and in vitro—amplifies type I interferon signaling through a mechanism dependent on ROS, thereby exerting pro-inflammatory effects [35]. Previous studies have established that ACOD1-derived itaconate inhibits succinate dehydrogenase (SDH) activity, suppressing succinate oxidation and promoting succinate accumulation in LPS-activated macrophages. This metabolic rewiring ultimately reduces the oxygen consumption rate (OCR) and limits the production of ROS [36]. Conversely, beyond its anti-inflammatory role, activation of the ACOD1 pathway can also potentiate inflammatory responses. For instance, LPS (or IFN-β) triggers ACOD1-dependent ROS generation, subsequently activating STAT1 and STAT3 [37]. There is further evidence that high levels of ACOD1 promote HSP70 degradation in a non-catalytic manner, leading to lysosome-dependent cell death [38]. Notably, our current study reveals that *Acod1* deficiency significantly alleviates ROS production in cells co-treated with LPS and 4-OI. This finding provides compelling evidence that ACOD1 promotes ROS generation and mitochondrial dysfunction in this model through a mechanism independent of endogenous itaconate production. This process transforms macrophages from immune sentinels into drivers of uncontrolled inflammation and tissue damage, underscoring the pathogenic role of metabolic overload in sepsis progression.

We further identified the AIM2-mediated PANoptosome as the scaffold responsible for 4-OI-induced death. As a member of the p200 protein family, AIM2 consists of an HIN domain and a pyrin domain (PYD). Under basal conditions, AIM2 is maintained in an autoinhibited, inactive state through intramolecular interactions between its own HIN and PYD domains. However, upon the presence of specific cytosolic stimuli, AIM2 undergoes a conformational change that exposes its PYD. This exposed PYD subsequently interacts with the PYD of the adaptor protein ASC, thereby driving ASC oligomerization [24]. We found that 4-OI promotes AIM2-ASC interaction and the activation of PANoptosis-associated protein. Crucially, the survival advantage observed in *Aim2*^*–/–*^ septic mice and the reduction of tissue-level PANoptosis-associated markers confirm that 4-OI activates the AIM2-PANoptosome axis to drive terminal organ failure. However, the results were based on the AIM2 *E2a-Cre* system, which obscures the potential contribution of other cell types. In future studies, we will employ the *Lyz2-Cre* system to further investigate this issue.

At the molecular level, we demonstrated that itaconate covalently alkylates AIM2 via Michael addition at the Cys113 residue. Intriguingly, this specific alkylation sterically shields AIM2 from physical interaction with the E3 ubiquitin ligase WWP1. Under physiological conditions, WWP1 restricts basal levels of AIM2 through K48- and K63-linked polyubiquitination and subsequent proteasomal degradation. By blocking this clearance pathway, Cys113 itaconation markedly prolongs the half-life of AIM2, leading to its pathological accumulation. This post-translational “stability remodeling” provides the necessary scaffold for the explosive assembly of the ASC-dependent macromolecular PANoptosome, ultimately dictating inflammatory cell death in macrophages. The in vivo relevance of this newly identified itaconate-AIM2 axis was rigorously validated using genetic intervention models. Point mutation of the alkylation site (C113A) completely abrogated itaconate-mediated AIM2 stabilization and ASC recruitment, rendering reconstituted macrophages highly resistant to 4-OI-induced PANoptosis. More importantly, global *Aim2* deletion dramatically protected mice from 4-OI-exacerbated polymicrobial sepsis, significantly attenuating systemic cytokine storms, lipid peroxidation, and extensive structural damage in critical organs (lungs, liver, and kidneys). While in vitro lentiviral models robustly proved the biochemical mechanism, generating an *Aim2* C113A knock-in mouse remains necessary. These rescue experiments not only establish AIM2 alkylation as an absolute prerequisite for the pro-sepsis lethality of itaconate but also highlight the AIM2-PANoptosome axis as a promising therapeutic target for mitigating sepsis-induced multi-organ dysfunction.

In conclusion, our study fundamentally shifts the paradigm regarding the ACOD1-itaconate axis from a unilateral anti-inflammatory mechanism to a complex, phase-specific regulator of immune fate. We prove that metabolic accumulation in late sepsis is not a protective relic, but rather a toxic trigger that mechanically locks AIM2 into a stabilized conformation via Cys113 alkylation, thereby igniting PANoptosome assembly and lethal inflammation. Deciphering this mechanism not only expands the frontiers of immunometabolism and post-translational modifications but also emphasizes the critical need for stage-specific precision medicine in managing life-threatening infectious diseases.

## Experimental model and study participant details

### Patient samples

Peripheral blood mononuclear cells (PBMCs) from healthy controls (*n* = 7) and 3-day post-sepsis patients (*n* = 7) were collected from Shanghai General Hospital between January 2024 and October 2024 (see Supplementary Table 1). The collection of samples was approved by the Ethics Committee of Shanghai General Hospital (2019KY033), and written informed consent was obtained. Sepsis was established according to the Third International Consensus Definitions for Sepsis and Septic Shock (Sepsis-3) [39]. PBMCs were isolated from human blood using a lymphocyte separation medium (Axis-Shield). Fresh whole blood (20 mL) was diluted in PBS (20 mL), layered on Lymphoprep (20 mL), and spun for 20 min at 2000 rpm. The PBMCs were isolated from the middle layer.

### Animal studies

C57BL/6J (wild type) mice were purchased from Legen Biotechnology Co., Ltd (Shanghai, China). *Acod1*^*–/–*^, *Aim2*^*–/–*^, *Zbp1*^*–/–*^, *Mefv*^*–/–*^, and *Nlrp3*^*–/–*^ mice were generated by Cygen Biosciences (Suzhou, China). All mice were bred and housed under specific pathogen-free conditions at the Animal Resource Center at Shanghai General Hospital and were backcrossed to the C57BL/6 J background for at least 10 generations. Mice were maintained at 24 ± 2 °C and 40–70% relative humidity with a 12-h light/dark cycle with lights on at 8:00 am and were fed standard chow. Food and water were accessible ad libitum. Male 6- to 8-week-old mice were used in vivo, and both male and female 6- to 12-week-old mice were used in vitro in this study. Animal studies were conducted under protocols approved by the Animal Use Committee of Shanghai General Hospital, Shanghai Jiao Tong University (No. 2023AW055).

### Murine bone marrow-derived macrophage cells (BMDMs) isolation and culture

Bone marrow was obtained by flushing RPMI-1640 (Gibco) medium through the tibia and femur of 6- to 8-week-old C57BL/6J mice with a 23-gauge needle. Subsequently, the bone marrow was resuspended in red cell lysis buffer (C3702, Beyotime) for 2 min before being resuspended in an equal volume of RPMI-1640 medium, passed through a 200-μm cell strainer and centrifuged. The final harvested cells were plated in RPMI-1640 medium containing 10% fetal bovine serum (FBS), 1% penicillin and streptomycin (C100C5, NCM), and 10 ng/mL recombinant macrophage colony-stimulating factor (M-CSF, 315-02, Peprotech) at 37 °C in a 5% CO_2_ incubator for 6 days.

### Peritoneal macrophage cells (PMs) isolation and culture

C57BL/6J mice were intraperitoneally injected with 2–3 mL of sterile 5% thioglycollate medium (T9032, Sigma-Aldrich) on two consecutive days. On day 4, mice were euthanized, and cells were collected by peritoneal lavage with cold PBS. The peritoneal lavage fluid was centrifuged for 5 min at 1000 rpm, and the cell pellet was resuspended in red cell lysis buffer for 2 min. Cells were then seeded in DMEM (Gibco) supplemented with 10% FBS, and 1% penicillin and streptomycin. Twenty-four h after isolation, the cells were subjected to the indicated treatments.

### In vitro experimental design and compound treatment

All in vitro experiments included appropriate vehicle controls. 4-octyl itaconate (4-OI; HY-112675, MedChemExpress) and dimethyl itaconate (DMI; 592498, Sigma-Aldrich) were dissolved in DMSO, whereas unmodified itaconate (ITA; I29204, Sigma-Aldrich) was dissolved in sterile double-distilled water (ddH2O) as a vehicle control. RAW264.7 macrophages, BMDMs, and PMs were washed with PBS, switched to a serum-free medium, and subjected to the following treatments for 24 h: (1) Concentration gradient: Cells were treated with 4-OI (50-1000 μM), DMI (50-1000 μM), or ITA (0.625-10 mM), with media pH adjusted to 7.4 using 1 M NaOH. (2) LPS priming: BMDMs were pre-stimulated with LPS (100 ng/mL; L2630, Sigma-Aldrich) for 3 h before 4-OI exposure to establish an inflammatory priming model.

## Materials and methods

### Metabolite profiling by LC-MS

TCA metabolites in PBMCs were extracted using a methanol/water mixture and quantified via LC-MS/MS on a QTRAP 6500+ system (SCIEX) at APExBIO Technology (China). Chromatographic separation was achieved on an ACQUITY UPLC BEH Amide column. Metabolites were ionized in both positive and negative ESI modes. Absolute quantification was performed using external standard calibration curves (correlation coefficient *R* > 0.99) for key standards, including itaconate, pyruvate, and succinate. Separately, the metabolomic profiling of BMDMs was performed by Shanghai Luming Biological Technology Co., Ltd. Intracellular metabolites were separated using an ACQUITY UPLC HSS T3 column and analyzed on a Q-Exactive mass spectrometer (Thermo Fisher Scientific) in both positive and negative ESI modes.

### mRNA-seq analysis

Total RNA was extracted from BMDMs using the Universal RNA Extraction CZ Kit (RNC643, ONREW). RNA quantity and quality were assessed by Qubit and denaturing agarose gel electrophoresis. RNA libraries were prepared with the VAHTS Universal V8 RNA-seq Library Prep Kit (NR605-0, Vazyme) and sequenced on the Illumina NovaSeq 6000 platform (150-bp paired-end reads). Raw reads were processed by Skewer for adapter trimming, and quality was checked with FastQC. Clean reads were aligned to the mouse genome (mm10) using STAR (allowing one mismatch). Gene expression levels were quantified by StringTie, and differential expression analysis was performed using DESeq2.

### Murine sepsis model establishment and pharmacological intervention

The mouse model of sepsis was established through cecal ligation and puncture (CLP), which is currently considered the gold standard in sepsis studies [40]. Male C57BL/6J mice were anesthetized with inhaled sevoflurane (2–4% mixed with air) and subjected to midline laparotomy. The cecum was ligated 1 cm distal to the ileocecal valve using a non-absorbable 4-0 silk suture while preserving intestinal continuity. The transmural puncture was created with a 22-G needle, followed by gentle fecal extrusion to ensure patency. After surgical closure, the mice received subcutaneous pre-warmed saline (5 mL/100 g body weight) and buprenorphine (0.05 mg/kg). Sham-operated controls underwent identical procedures, excluding the ligation and perforation steps. Mice in the CLP + 4-OI group received intraperitoneal injections of 4-OI (50 mg/kg) at 2, 24, 48, and 72 h post-CLP [41], whereas mice in the 4-OI control group were administered equivalent drug doses without CLP induction.

### Tissue histopathology

Following perfusion with heparinized PBS (6.25 IU/mL), lung, liver, and kidney tissues were fixed in 4% paraformaldehyde, paraffin-embedded, and sectioned at 4-μm thickness. Sections were stained with hematoxylin and eosin (H&E) for histological evaluation, and digital images were acquired using the KFBIO KF-PRO-120 digital pathology slide scanner. The severity of lung injury was evaluated according to a well-established semi-quantitative scoring system as previously described [42]. Liver damage was graded as follows: 0, no damage; 1, mild (cytoplasmic vacuolation, focal nuclear pyknosis); 2, moderate (sinusoidal dilation, cytoplasmic vacuolation, blurred cell boundaries); 3, moderate-to-severe (coagulative necrosis, marked sinusoidal dilation, erythrocyte extravasation, eosinophilic change, neutrophil margination); 4, severe necrosis (architectural loss, hepatic cord disruption, hemorrhage, massive neutrophil infiltration). Renal injury was scored based on the percentage of affected area: 0, no injury; 0.5, <10%; 1, 10–25%; 2, 25–50%; 3, 50–70%; 4, ≥ 70%.

### Lung wet/dry weight ratio

The fresh left lung was excised and immediately weighed to obtain the wet weight. The tissue was then subjected to complete dehydration in a constant-temperature oven at 60 °C for 72 h before dry weight measurement. The wet/dry weight ratio was subsequently determined to quantify pulmonary edema severity.

### ELISA and biochemical assays

Plasma samples from mice were analyzed via IL-1β (88-7013, ThermoFisher), TNF-α (88-7324, ThermoFisher), and IL-6 (88-701364, ThermoFisher) ELISA kits. Tissue lipid peroxidation was measured using Malondialdehyde (MDA, A003-1-2) Assay Kits. Hepatic and renal function markers, including tissue aspartate aminotransferase (AST, C010-2-1), alanine aminotransferase (ALT, C009-2-1), blood urea nitrogen (BUN, C013-2-1), and creatinine (Cr, C011-2-1), were quantified using corresponding assay kits (Nanjing Jiancheng Bioengineering Institute). The concentration of AIM2 in cell culture supernatants was detected using a Mouse AIM2 ELISA Kit (U96-1618E, YOBIBIO).

### Cell culture

The Raw264.7 and HEK293T cell lines were cultured in DMEM supplemented with 10% FBS, and 1% penicillin-streptomycin at 37 °C in a 5% CO_2_ incubator.

### Immunoblotting

Cells and tissues were washed with 1× PBS and lysed in RIPA lysis buffer (WB3100, NCM) containing protease inhibitors. Protein concentrations were determined using the BCA Protein Assay Kit (WB6501, NCM). Cell lysates were separated by 10% or 12.5% SDS-PAGE and transferred to polyvinylidene difluoride (PVDF) membranes via wet transfer. The membranes were blocked with Blot Blocking Buffer (P30500, NCM) for 15 min at room temperature and incubated with the primary antibodies and secondary HRP-conjugated antibodies at a 1:5000 dilution. Membranes were visualized using Omni-ECL™ (SQ201, Epizyme Biotech) on a GelDoc system (Bio-Rad). Images were analyzed with ImageLab (Bio-Rad) and ImageJ software. Details of the antibodies used are shown in Supplementary Table 4.

### Cell viability assay

The CCK-8 kit was used to assess the effects of 4-OI, DMI, and ITA on cell viability. Raw264.7 cells, BMDMs, and PMs were seeded in 96-well plates and treated with various doses of 4-OI, DMI, and ITA for 24 h. The cells were then incubated with a 10% CCK-8 solution in the medium at 37 °C for 2-3 h before measuring the absorbance at 450 nm using a Varioskan Flash microplate reader (Synergy™ HTX).

### Cell death assay

Cells were cultured in six-well plates and treated with different doses of 4-OI or a mixture of LPS (100 ng/mL) and 4-OI (500 μM) for 24 h. SYTOX Green (1:3000, S7020, Invitrogen) was added to the medium, and cells were incubated at 37 °C for 15 min. SYTOX staining was detected by immunofluorescence. Images were acquired using a Leica SP8 confocal microscope, and quantitative analysis was performed using ImageJ software.

### Immunofluorescence staining

Immunofluorescence staining was performed as described previously [43]. Briefly, following treatment, BMDMs were fixed in 4% paraformaldehyde for 10 min and permeabilized for 10 min in PBS containing 0.5% Triton X-100. Cells were subsequently blocked in PBS with 1% bovine serum albumin (BSA) for 1 h, followed by overnight incubation with the corresponding primary antibodies at 4 °C. Alexa Fluor 488-conjugated anti-pMLKL (37333S, Cell Signaling Technology) or AIM2 (20590-1-AP, Proteintech), Alexa Fluor 555-conjugated anti-cleaved-Caspase-3 (373730, Santa Cruz Biotechnology), or Alexa Fluor 647-conjugated anti-ASC (Ab175449, Abcam) were incubated with corresponding antibodies (1:500) for 2 h under the coverslips. DAPI mounting media (P36931, Invitrogen) was used to counterstain the nuclei. Images were obtained by a Leica SP8 confocal microscope equipped with a 63 × Oil objective.

### RNA extraction and qPCR

Trizol Reagent (T9108, Takara) was used to collect and extract total RNA from tissues or cells. Eluted RNA was measured with a Nanodrop (Thermo Fisher Scientific), and each RNA sample was quantified and diluted to the lowest possible yield before reverse transcription. The SuperScript®III Kit (18080093, Thermo Fisher Scientific, 18080093) was used to generate cDNA according to the manufacturer’s instructions. qRT-PCR was performed using SYBR Green Master Mix on QuantStudio 6 Flex system (Applied Biosystems). The data were analyzed using the ΔΔCT method to compare relative expression levels. Details of the primers used for the qRT-PCR analysis are shown in Supplementary Table 5.

### Assessment of mitochondrial function and ROS production

To evaluate mitochondrial mass and morphology, cells were incubated with MitoTracker™ Red for 30 min at 37 °C in the dark. Following incubation, the cells were washed twice with PBS to remove unbound dye. Mitochondrial membrane potential (ΔΨm) was assessed using the JC-1 fluorescent probe. Briefly, treated cells were harvested, washed, and stained with the JC-1 working solution for 20 min at 37 °C according to the manufacturer’s instructions. The transition of JC-1 from red fluorescence (J-aggregates, representing intact membrane potential) to green fluorescence (J-monomers, representing depolarized mitochondria) was measured to evaluate the extent of mitochondrial depolarization. For the detection of ROS, cells were incubated with the fluorescent probe DCFH-DA Red for ROS in a serum-free medium for 30 min at 37 °C in the dark. After the respective staining and washing procedures, the fluorescence intensities of MitoTracker, JC-1, and ROS probes were immediately quantified using a fluorescence microscope.

### ASC oligomerization assay

The ASC oligomerization assay was performed as previously described with minor modifications [28]. Briefly, BMDMs were plated in 6-well plates and primed with LPS (100 ng/mL) for 3 h before adding 4-OI (500 μM). The cells were washed twice with PBSand detached by scraping in TBS containing 0.5% Triton X-100. After centrifugation, the cell pellet was resuspended in ice-cold lysis buffer and lysed by sonication. Cells were centrifuged at 6000 × *g* for 15 min to remove a large number of nuclei, then 20 μl of the lysate was saved as the input, and the remaining lysate was treated with 4 mM disuccinimidyl suberate (DSS) for 30 min at room temperature. After centrifugation, the pellets were resuspended in 25 μl of loading buffer and boiled at 100 °C for 5 min. The cross-linked proteins were separated by 10% SDS–PAGE and analyzed for ASC oligomerization by immunoblotting with a mouse monoclonal anti-ASC antibody (sc-514414, Santa Cruz Biotechnology).

### Proximity-ligation assay

The Proximity Ligation Assay (PLA) Kit (DUO96010 and DUO96000, Sigma-Aldrich) tool for visualizing protein-protein interactions was used to examine the interaction between ASC and AIM2, as well as between FLAG-tagged AIM2 and FLAG-tagged AIM2 mutants. Briefly, 10,000 BMDMs were cultured on 12-mm coverslips (Thermo Fisher Scientific) in 24-well plates. Cells were fixed with 4% formaldehyde for 10min, permeabilized with 0.5% Triton X-100 for 10min, and then incubated overnight at 4 °C with primary antibodies, a pair of different species targeting AIM2 (rabbit monoclonal) and ASC (mouse monoclonal). In situ PLA was performed according to the manufacturer’s instructions. Protein-protein interactions were observed using a Leica SP8 confocal microscope and quantified using ImageJ software.

### Quantification of itaconation targets with ITalk (containing metabolic labeling, protein identification and site identification)

Frozen cells were lysed and centrifuged, and the protein concentration of the supernatants was adjusted to 2 mg/mL (BCA assay). Click chemistry labeling was performed using TAMRA-azide or Biotin-DADPs-azide with CuSO₄/BTTAA and sodium ascorbate (1 h, 25 °C). Labeled proteins were resolved by SDS-PAGE and visualized by fluorescence or Coomassie staining. Biotinylated proteins were enriched with streptavidin beads, washed, and digested on-bead with LysC/trypsin. For site-specific analysis, modification-containing peptides were eluted with 0.1% TFA. Peptides were analyzed by LC-MS/MS on an EASY-nLC 1200 coupled to Q Exactive HF-X. MS1 scans were at 60,000 resolution, and the top 20 precursors were selected for HCD fragmentation with MS2 at 15,000 resolution.

### In situ metabolic tagging and click chemistry profiling of endogenous AIM2

RAW264.7 and BMDMs were cultured to 70% confluency in 15 cm dishes, stimulated with LPS (10 or 100 ng/mL) for 12 h, and then metabolically labeled with 100 μM ITalk for 12 h. Cells were washed with PBS, pelleted, and stored at -80 °C. The pellets were lysed in PBS containing protease inhibitors by sonication and centrifuged (20,000 × *g*, 30 min, 4 °C), after which the protein concentration was determined by a BCA assay. A 50 μL aliquot (2 mg/mL) was saved as the input. Click chemistry was performed using CuSO₄ (1 mM), TBTA (100 μM), Biotin-Azide (100 μM), and TCEP (1 mM) for 1 h at room temperature (RT). After centrifugation (8000 × *g*, 5 min, 4 °C) and methanol washes, the pellets were resuspended in 1.2% SDS/PBS, sonicated, heated (90 °C, 5 min), and diluted to 0.2% SDS. The Samples were incubated with streptavidin beads (100 μL, 3 h, 29 °C), washed with PBS and water, and eluted with loading buffer (95 °C, 10 min). The input and elution fractions were analyzed by Western blot for AIM2.

### Identification of AIM2 modification sites by ITalk

HEK293T cells were cultured in 15-cm dishes to 70% confluence and then transfected with pCMV-3×FLAG-AIM2 plasmids (WT or mutants) using EZ trans (AC04L051, Shanghai Life iLab Biotech Co., Ltd.) for 24 h. Subsequent metabolic labeling was achieved through ITalk click chemistry under optimized catalytic conditions following the protocol detailed in the previous section. Input and elution samples were separated on 10% SDS-PAGE gels, and FLAG-AIM2 was detected by Western blot using anti-FLAG antibody.

### AIM2 structural modeling

The full-length protein structure of AIM2 was predicted by ROBETTA’s website (https://robetta.bakerlab.org). Protein structural accuracy was validated via the UCLA SAVES server (https://saves.mbi.ucla.edu/), confirming high structural reliability (with 91.1% of residues located in the most favored regions). For the preparation of the small ligand ITalk and AIM2 protein, use AutoDock Tools 1.5.2 to assign all hydrogens, add Gasteiger charges and set up rotatable bonds. AutoDock was used to perform covalent docking. PyMol was utilized for the final 3D visualization.

### Statistical analysis

Statistical analyses were conducted with GraphPad Prism v8.4.3. Experimental replicates are detailed in the corresponding figure legends. Differences between two independent group were analyzed by two-tailed Student’s t-tests, while multiple comparisons were evaluated using two-way ANOVA with Tukey’s multiple comparisons test. Survival rates were assessed using the Kaplan-Meier method. Quantimetric data are expressed as mean ± SEM. Statistical significance was established at *P* < 0.05 throughout all analyses.

## Supplementary information

Supplementary Figs. and the Fig. legend

original images

Supplementary tables
