# Supplementary material for: The alkylation of AIM2 by itaconate mediates macrophage PANoptosis during sepsis
Source: Cell Mol Immunol. 2026 May 12;23(6):619–34. doi: 10.1038/s41423-026-01414-x (PMC13222358; doi:10.1038/s41423-026-01414-x)
Supplement: Supplementary file 1 — Supplementary Figs. and the Fig. legend [file 41423_2026_1414_MOESM1_ESM.docx]

**Supplementary figures and figure legends**


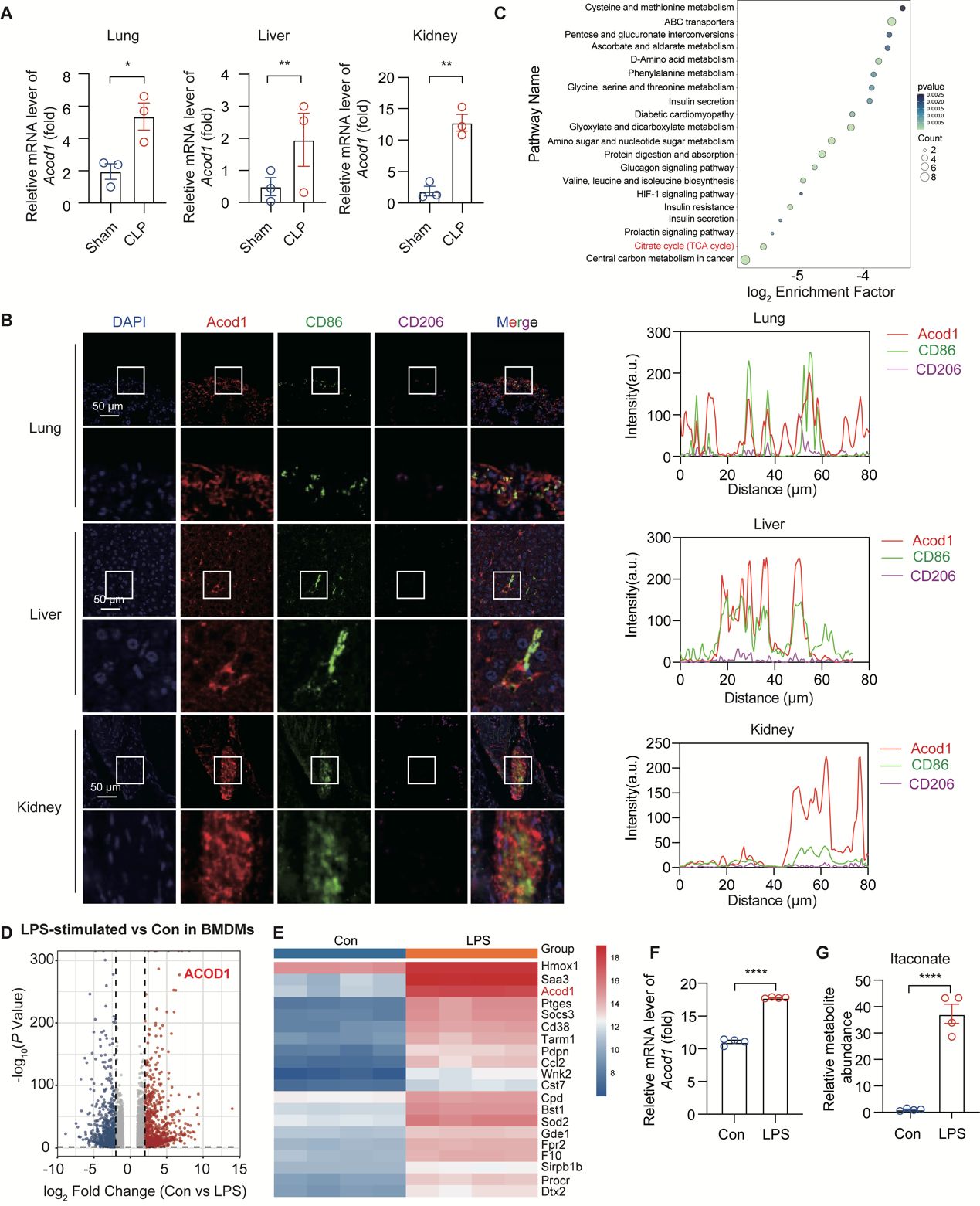


**Fig. S1** Persistently upregulated ACOD1-itaconate levels are closely associated with poor prognosis in septic patients. **A** qRT-PCR results showing mRNA expression of *Acod1* normalized to 18s in mice lung liver and kidney collected at 72 hours after CLP modelling. Data are representative of at least three independent experiments. **B** Immunofluorescence colocalization analysis of ACOD1 with CD86 (M1 macrophage marker) and CD206 (M2 macrophage marker) in the lung, liver, and kidney of septic mice. Images are representative of three independent experiments. **C** KEGG pathway enrichment analysis showing the top 20 upregulated metabolic pathways in PBMCs from healthy volunteers (n = 7) and 3-day post-sepsis patients (n = 7), analyzed by LC-MS. RNA-Seq analysis in BMDMs stimulated with LPS for 24 h. Volcano plot (**D**) highlighting significantly downregulated (blue dots) and upregulated (red dots) genes after LPS stimulation, and a heatmap (E) displaying the top 20 upregulated genes in the control (Con, n = 4) and LPS (n = 4) groups. **F** *Acod1* mRNA expression levels in BMDMs from Con (n = 4) and LPS (n = 4). **G** LC-MS quantification of intracellular itaconate levels in BMDMs from the Con (n = 4) and LPS (n = 4) groups. Data are displayed as mean ± SEM. Differences were considered statistically significant at **P* < 0.05, ***P* < 0.01, ****P* < 0.001, and *****P* < 0.0001.


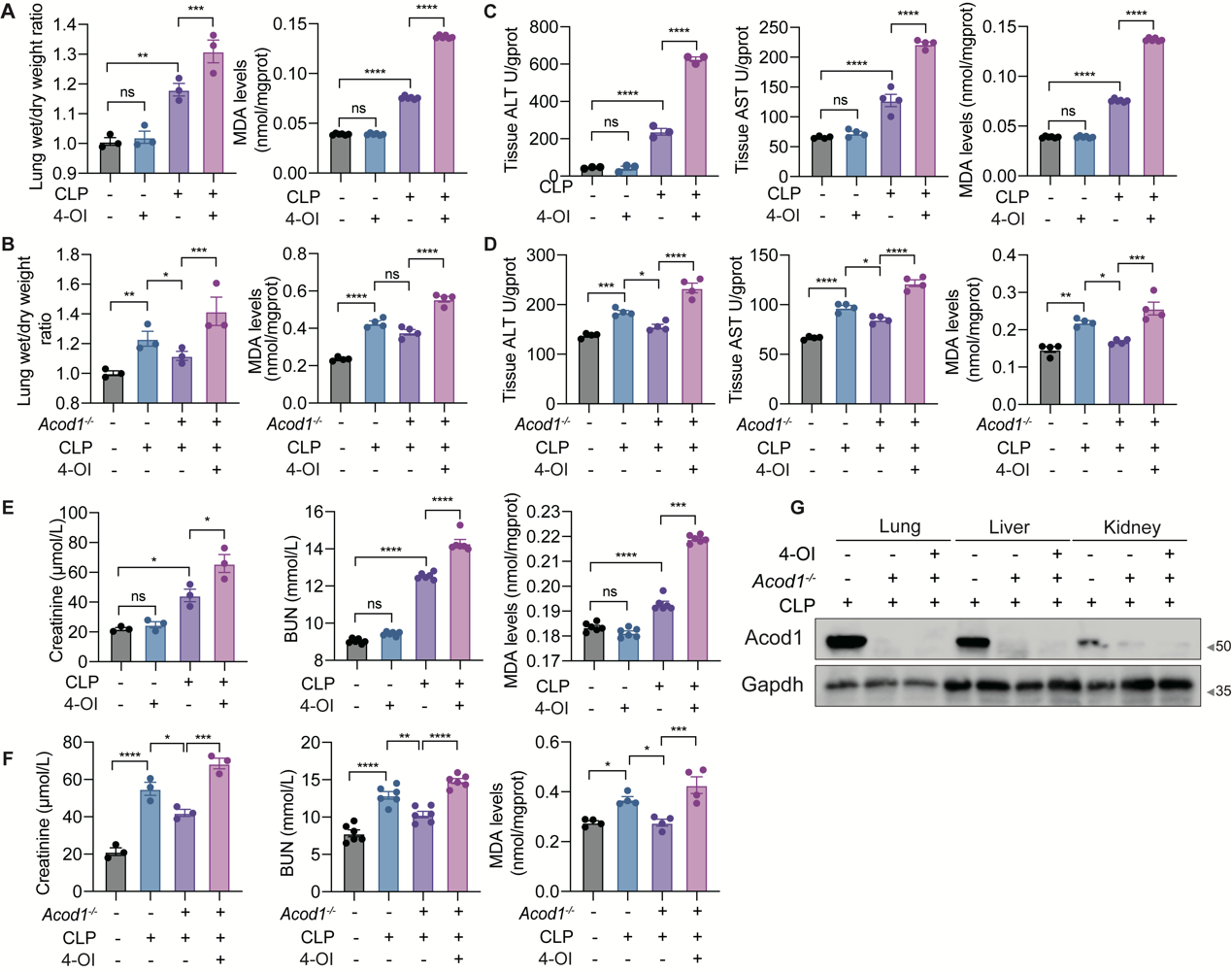


Fig. S2 The ACOD1-itaconate axis exacerbates systemic inflammation and multi-organ failure in sepsis. Assessment of pulmonary injury based on wet/dry weight ratios and MDA levels in WT (**A**) and *Acod1^-/-^* (**B**) mice. Evaluation of hepatic function and oxidative stress by serum ALT and AST, along with liver MDA levels, in WT (**C**) and *Acod1^-/-^* (**D**) mice. Evaluation of renal function and oxidative stress by serum Cr and BUN, along with kidney MDA levels in WT (**E**) and *Acod1^-/-^* (**F**) mice. **G** ACOD1 protein level of were analyzed by Western blotting in lung, liver, and kidney tissues from WT and *Acod1^-/-^* mice. Data are displayed as mean ± SEM. Differences were considered statistically significant at **P* < 0.05, **P < 0.01, ****P* < 0.001, and *****P* < 0.0001.


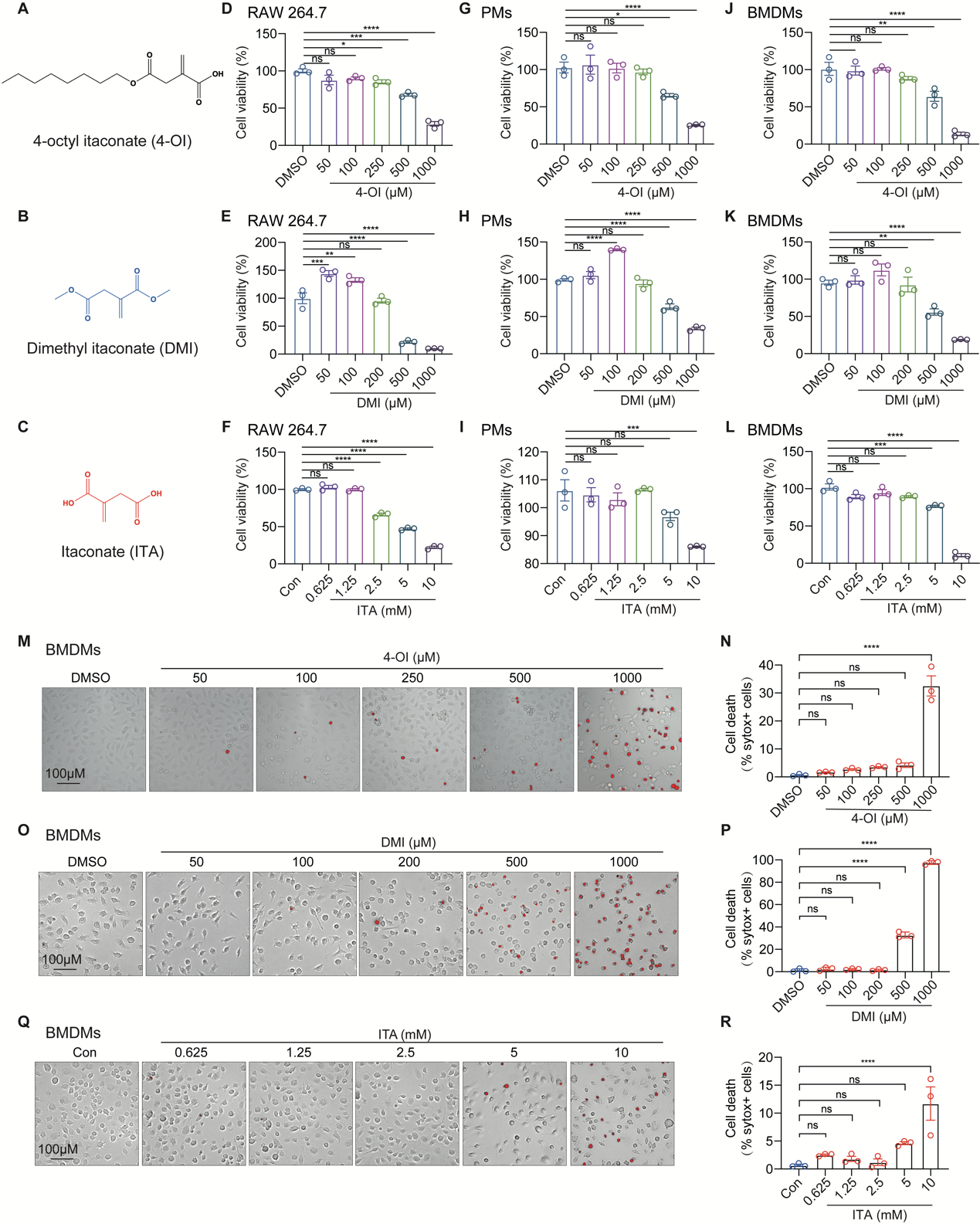


Fig. S3 High-dose itaconate induces macrophage cell death and drives pro-inflammatory responses during the immunosuppressive phase. Chemical structures of 4-octyl itaconate (4-OI; **A**), dimethyl itaconate (DMI; **B**) and unmodified itaconate (ITA; **C**). Cell viability assessed via CCK-8 assay (n = 3 per group) in RAW264.7 cells (**D**–**F**), PMs (**G**–**I**), and BMDMs (**J**–**L**) treated with the indicated concentrations of 4-OI (**D, G, J**), DMI (**E, H, K**), or ITA (**F, I, L**) for 24 h. Cell death evaluation in BMDMs treated with different concentrations of 4-OI (**M**), DMI (**O**), or ITA (**Q**) for 24 h, detected by SYTOX Green immunofluorescence and quantification of cell death. Scale bars, 100 μm. Quantification of the percentage of cells with sytox+ cells among the total cells in BMDMs with different concentrations of 4-OI (**N**), DMI (**P**) and ITA (**R**). Images are representative of three independent experiments (**M, O, Q**). Data are displayed as mean ± SEM. Differences were considered statistically significant at *P < 0.05, **P < 0.01, ***P < 0.001, and ****P < 0.0001.


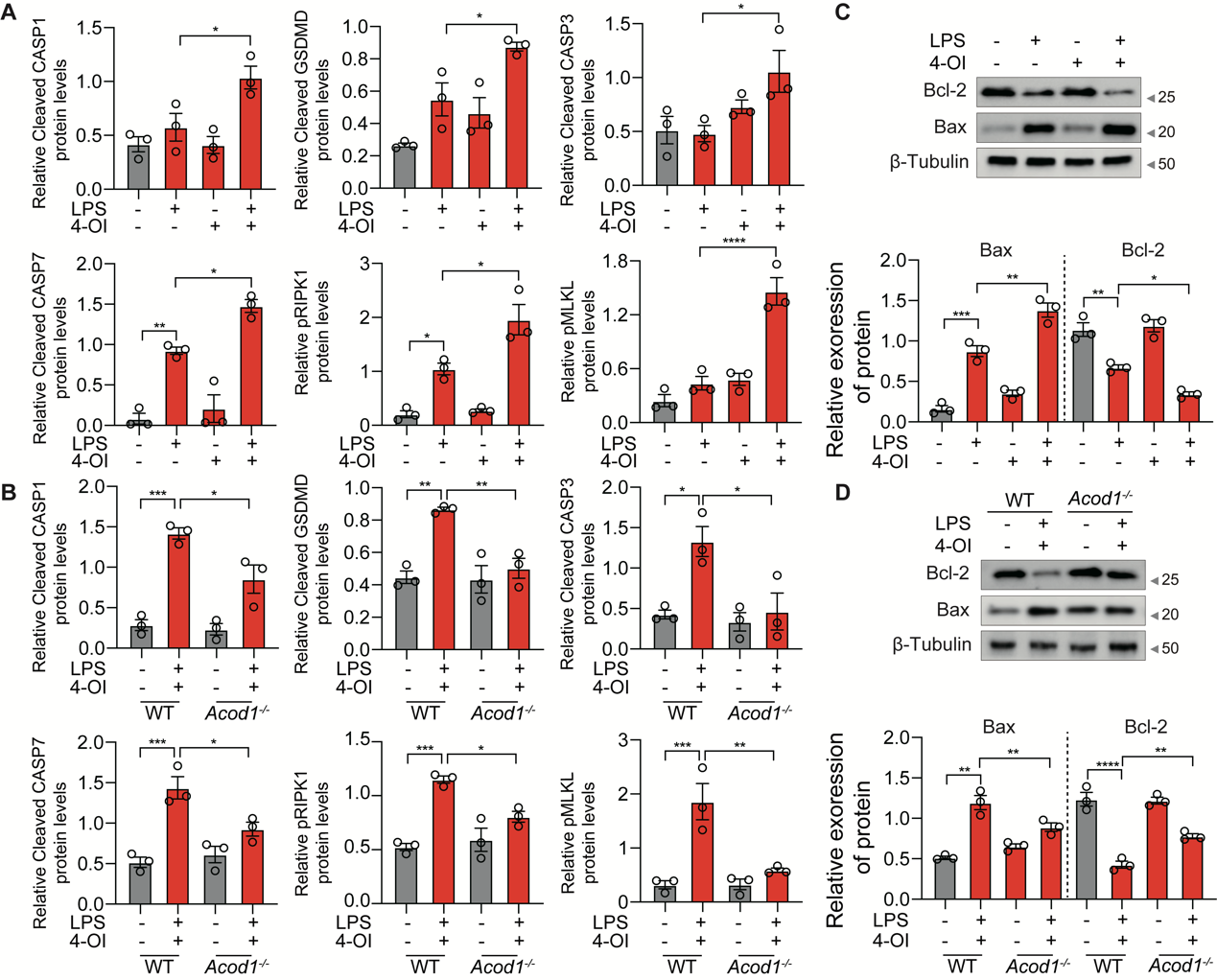


Fig. S4 Itaconate induces PANoptosis in LPS-activated macrophages. Quantification of cleaved-CASP1, cleaved-GSDMD, cleaved-CASP3, cleaved-CASP7, pRIPK1, and pMLKL protein expression in WT (**A**) and *Acod1^-/-^* (**B**) BMDMs. Immunoblotting analysis and quantification of BCL-2 and BAX protein levels in WT (**C**) and *Acod1^-/-^* (**D**) BMDMs. Data are displayed as mean ± SEM. Differences were considered statistically significant at **P* < 0.05, **P < 0.01, ****P* < 0.001, and *****P* < 0.0001.


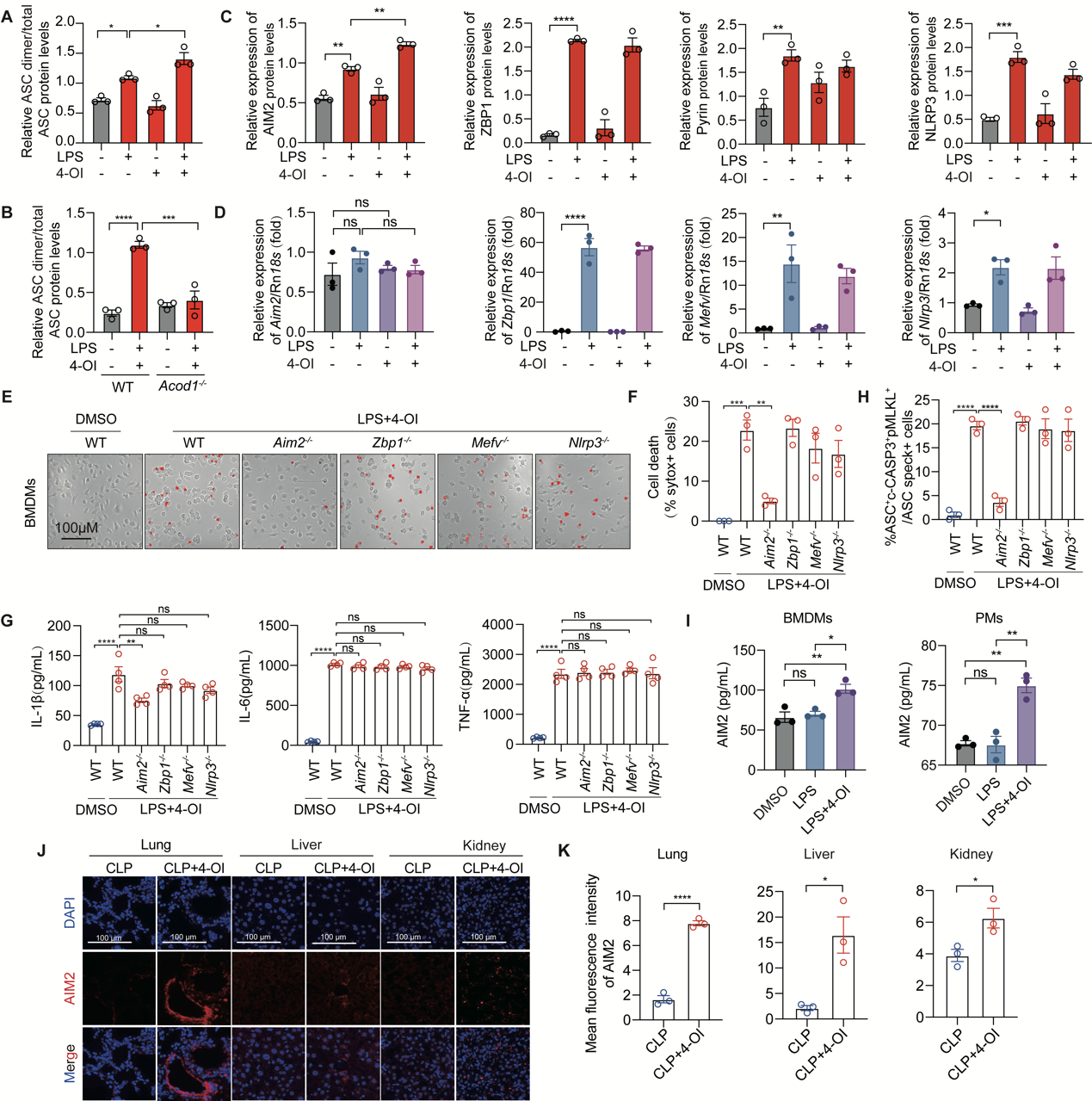


Fig. S5 Itaconate drives macrophage PANoptosis and exacerbates sepsis via AIM2-dependent PANoptosome assembly. Quantification of ASC dimers in WT (**A**) and *Acod1^-/-^* (**B**) BMDMs. **C** Quantification of AIM2, ZBP1, Pyrin and NLRP3 expression in WT BMDMs. **D** qPCR analysis of *Aim2*, *Zbp1*, *Mefv* and *Nlrp3* mRNA levels in BMDMs. **E** Cell death evaluation in WT, *Aim2^-/-^*, *Zbp1^-/-^*, *Mefv^-/-^*, and *Nlrp3^-/-^* BMDMs, measured by SYTOX Green uptake assay detected by immunofluorescence. Scale bar, 100 μm. **F** Quantification of the percentage of cells with sytox+ cells among the total cells in WT, *Aim2^-/-^*, *Zbp1^-/-^*, *Mefv^-/-^*, and *Nlrp3^-/-^* BMDMs. **G** ELISA quantification of IL-1β, IL-6 and TNF-α levels in the supernatants of WT, *Aim2^-/-^*, *Zbp1^-/-^*, *Mefv^-/-^*, and *Nlrp3^-/-^* BMDMs after LPS and 4-OI treatment (n = 4). **H** Quantification of the percentage of ASC^+^c-CASP3^+^pMLKL^+^ specks among total ASC speck^+^ cells. **I** ELISA quantification of AIM2 secretion in supernatants of BMDMs and PMs. **J** Immunofluorescence analysis of AIM2 in tissues of lung, liver, and kidney from CLP and CLP+4-OI groups. Scale bars, 100 μm. **K** Quantitative analysis of mean fluorescence intensity in **J**. Images are representative of at least three independent experiments (**E, J)**. Data are displayed as mean ± SEM. Differences were considered statistically significant at **P* < 0.05, **P < 0.01, ****P* < 0.001, and *****P* < 0.0001.


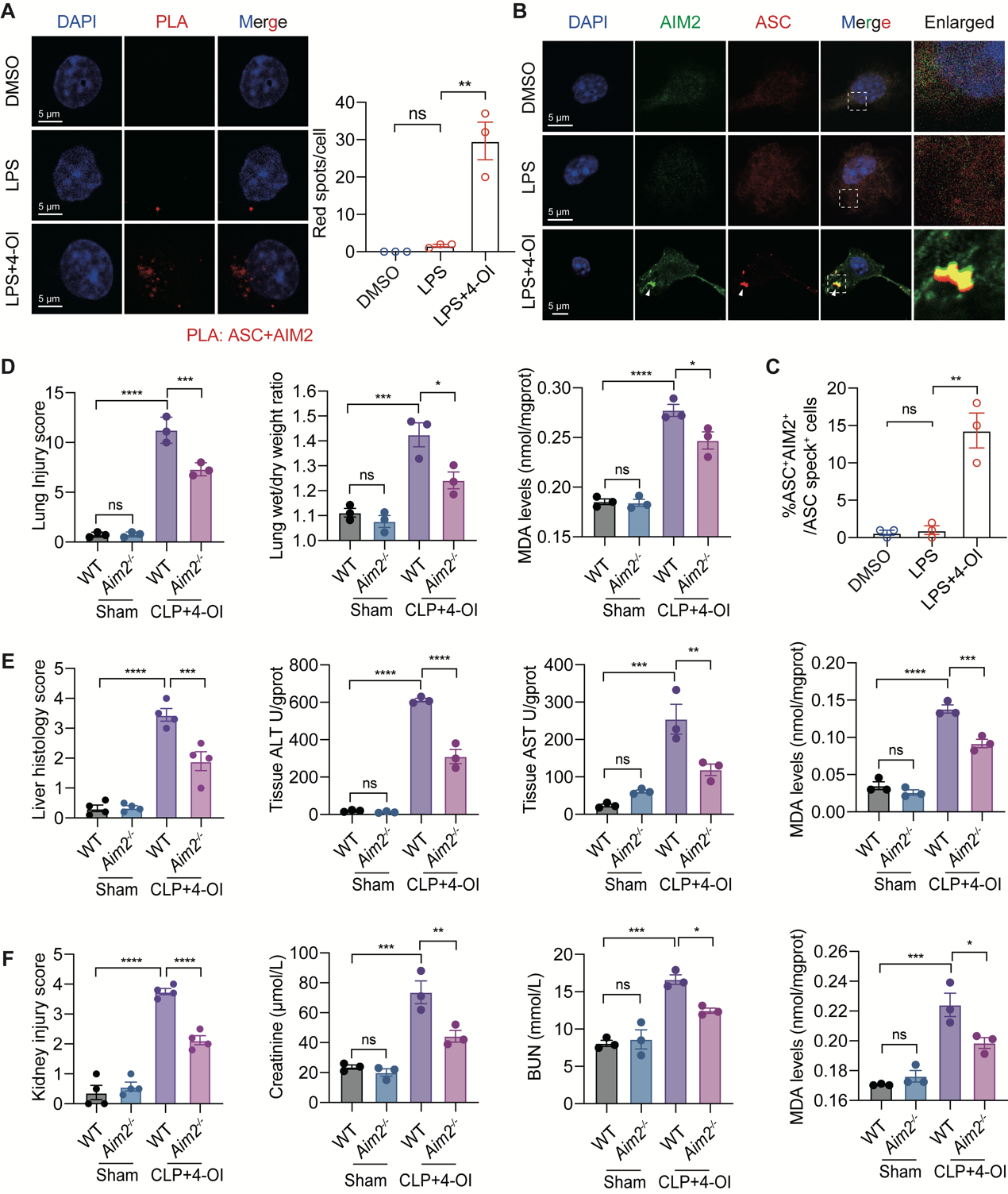


Fig. S6 Itaconate drives macrophage PANoptosis and exacerbates sepsis via AIM2-dependent PANoptosome assembly. **A** Proximity ligation assay (PLA) detecting physical associations between AIM2 and ASC (visualized as red fluorescent puncta) in BMDMs. Scale bars, 5 μm. **B** Immunofluorescence images of BMDMs at 24 h post-treatment with LPS or LPS+4-OI. Arrowheads indicate the ASC specks. **C** Quantification of the percentage of cells with ASC^+^AIM2^+^specks among the ASC speck^+^ cells. **D** Assessment of pulmonary injury based on lung injury scores, wet/dry weight ratios, and lung MDA levels in the indicated groups. **E** Evaluation of hepatic function and oxidative stress by liver injury score, serum ALT and AST, and liver MDA levels. **F** Evaluation of renal function and oxidative stress via kidney injury score, serum Cr and BUN, and kidney MDA levels. Images are representative of three independent experiments (**A, B**). Data are displayed as mean ± SEM. Differences were considered statistically significant at **P* < 0.05, **P < 0.01, ****P* < 0.001, and *****P* < 0.0001.


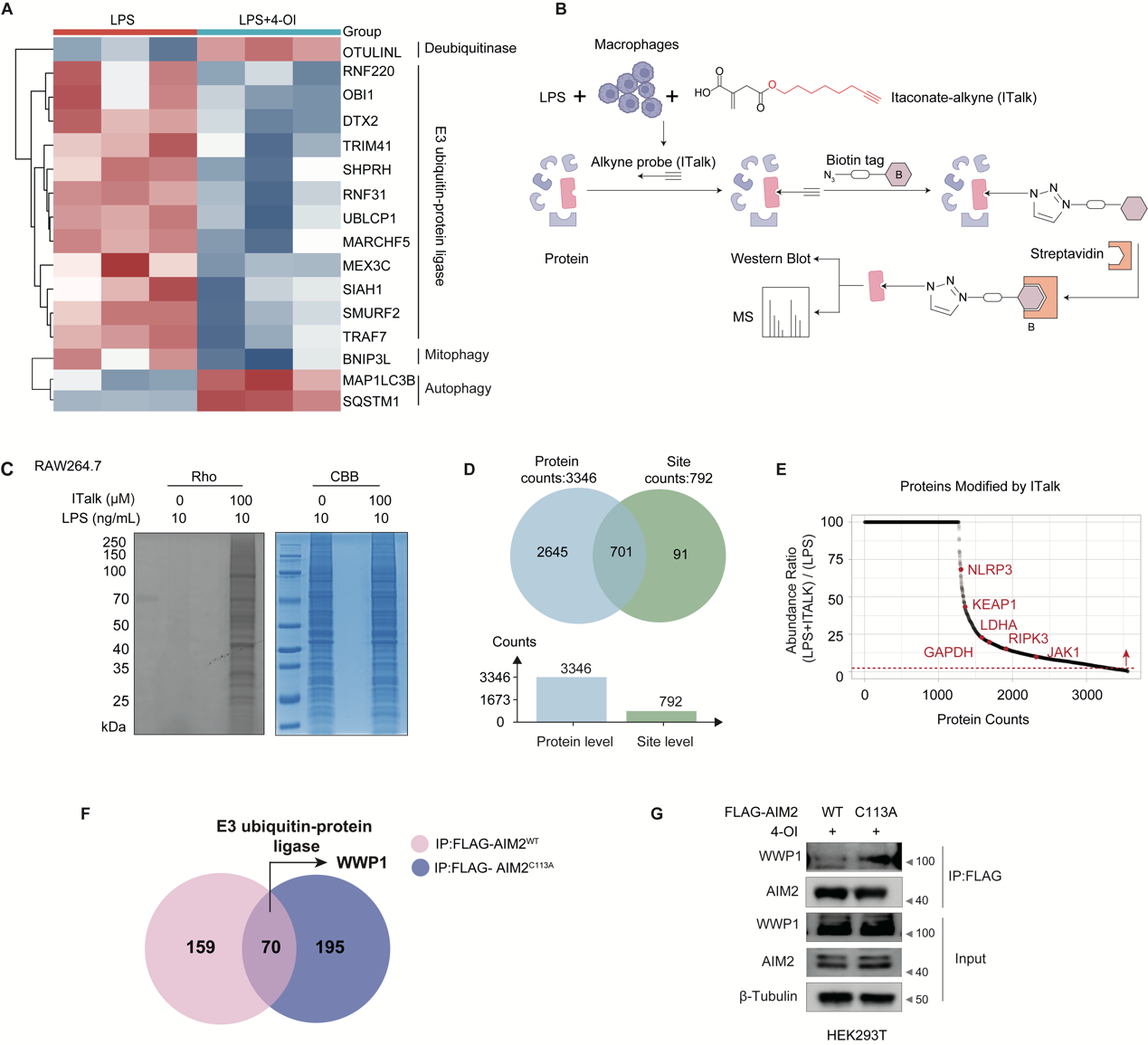


Fig. S7 Itaconate enhances AIM2 stability via alkylation at Cys113. **A** The HEK293T cells transfected with FLAG-AIM2 plasmids were stimulated with LPS (100 ng/mL) for 24 h, or pre-treated with LPS for 12 h followed by 4-OI (500 μM) for another12 h. Cell lysates were collected, and LC-MS analysis was employed to identify ubiquitin-proteasome system (UPS)- and autophagy-lysosomal pathways-related proteins enriched in the FLAG-AIM2 precipitates. **B** Schematic of click chemistry. RAW264.7 cells were treated with LPS (10 ng/mL) for 12 h and subsequently incubated with ITalk (100 μM) or DMSO for 12 hours. **C** The labeled lysates underwent a reaction with azide-rhodamine (Rho) via copper-catalyzed azide-alkyne cycloaddition (CuAAC), followed by the measurement of fluorescence intensity via in-gel fluorescence scanning. The uniformity of labeling was confirmed by Coomassie Brilliant Blue (CBB) staining. **D** Venn diagram analysis comparing the 792 proteins identified during site-specific identification with the 3,346 proteins identified during total protein identification. **E** Identification of hyper-sensitive itaconate targets by chemoproteomics. **F** HEK293T cells were transfected with AIM2-WT or AIM2-C113A plasmids. Cell lysates were immunoprecipitated with an anti-FLAG antibody, and the precipitated protein complexes were resolved by SDS-PAGE. Excised gel bands were subjected to LC-MS analysis to identify potential interacting proteins. **G** Co-immunoprecipitation and immunoblotting analysis of WWP1 in HEK293T cells expressing AIM2-WT or the AIM2-C113A mutant.


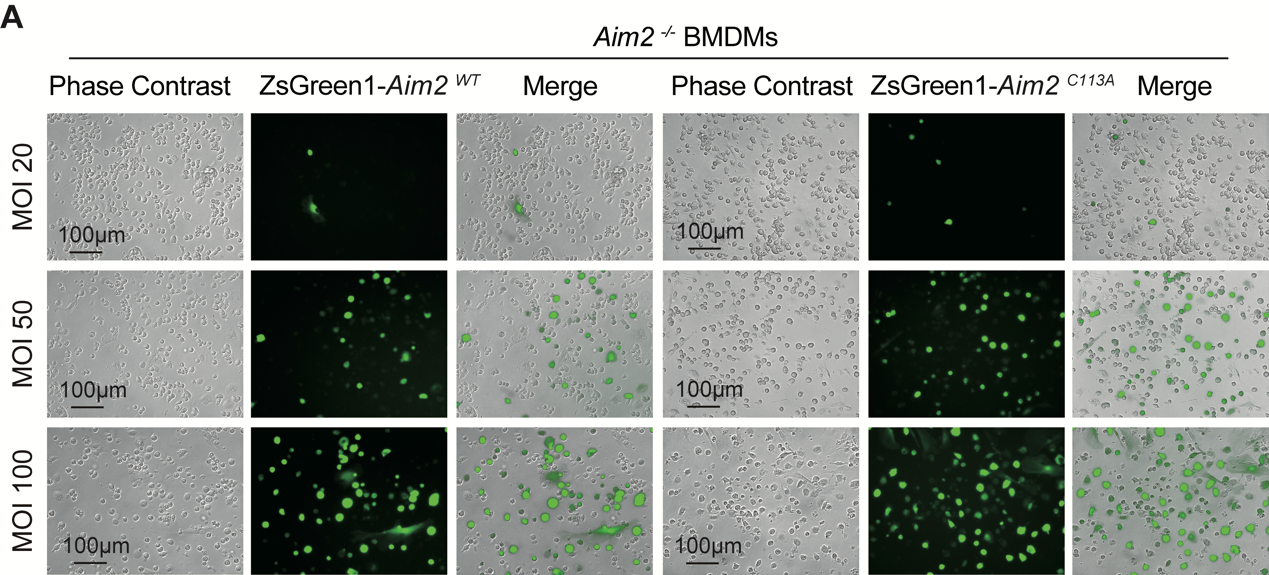


Fig. S8 Mutation of AIM2 at Cys113 alleviates itaconate-induced PANoptosome assembly and PANoptosis. **A** Representative fluorescence images validating the efficiency of BMDMs transduction at varying multiplicity of infection (MOI) numbers. Images are representative of three independent experiments.


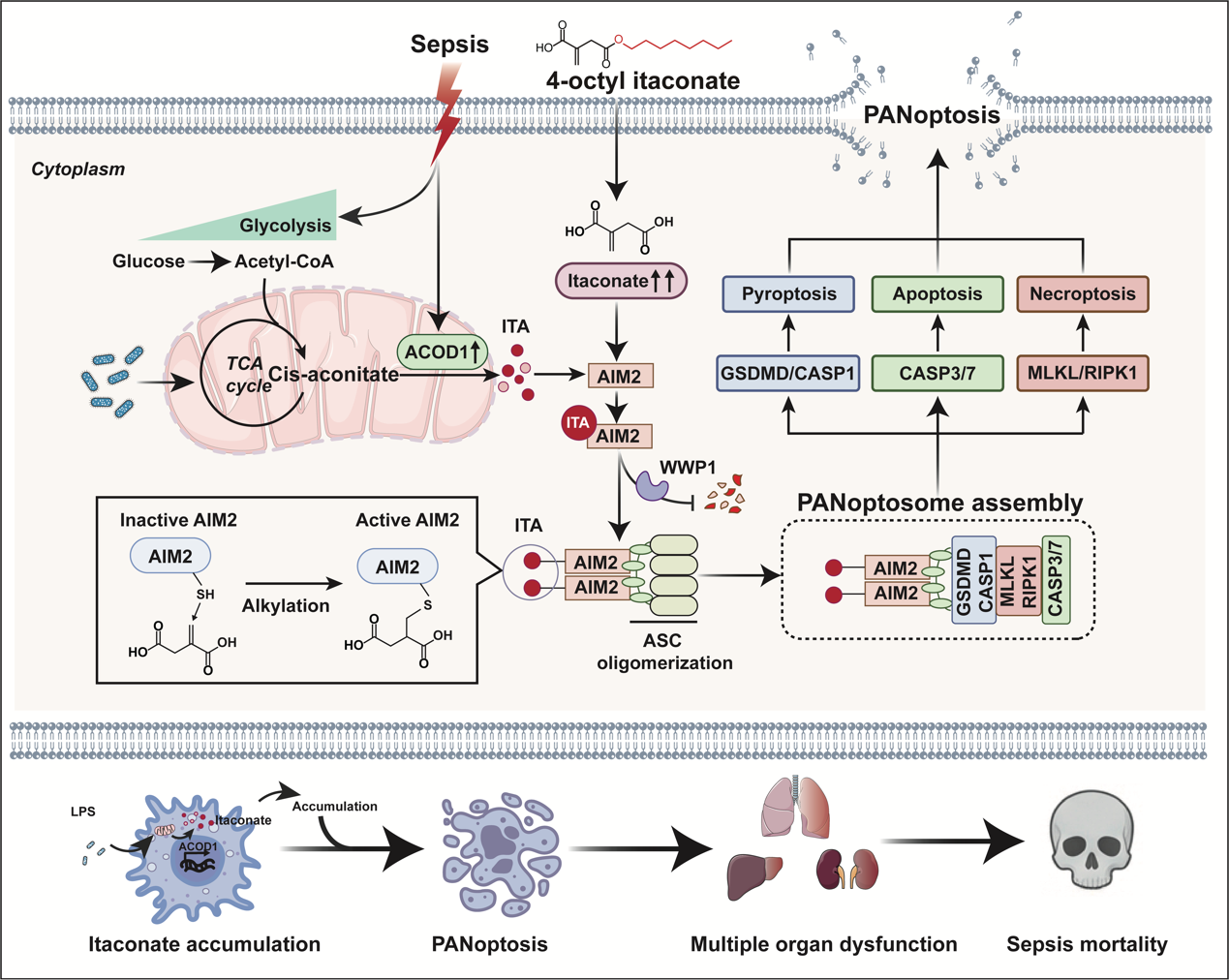


**Fig. S9** During sepsis, inflammatory stimulation promotes pathological itaconate accumulation in macrophages. By covalently alkylating the Cys113 residue of AIM2, itaconate stabilizes AIM2 protein and enhances AIM2-ASC interactions, thereby promoting PANoptosome assembly and subsequent PANoptosis. These pro-inflammatory and cytotoxic effects are effectively reversed by *Aim2* deficiency.
