# Supplementary material for: The alkylation of AIM2 by itaconate mediates macrophage PANoptosis during sepsis
Source: Cell Mol Immunol. 2026 May 12;23(6):619–34. doi: 10.1038/s41423-026-01414-x (PMC13222358; doi:10.1038/s41423-026-01414-x)

Figure. 1C

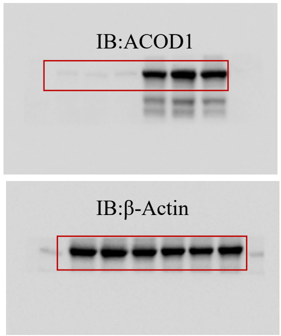

Figure. 1D

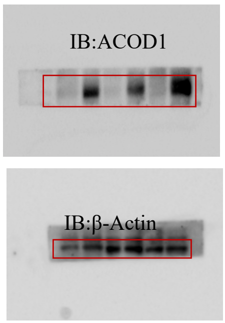

Figure. 1E

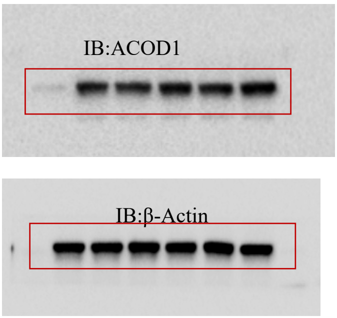

Figure. 1F

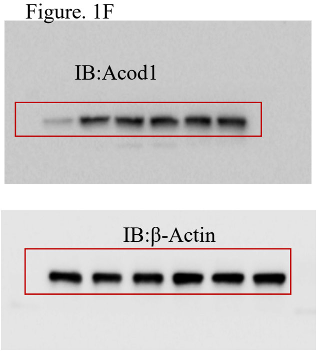

Figure. 4A

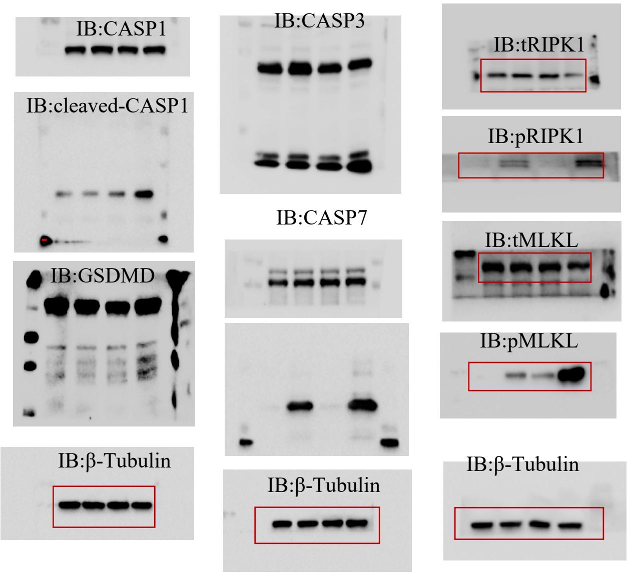

Figure. 4B

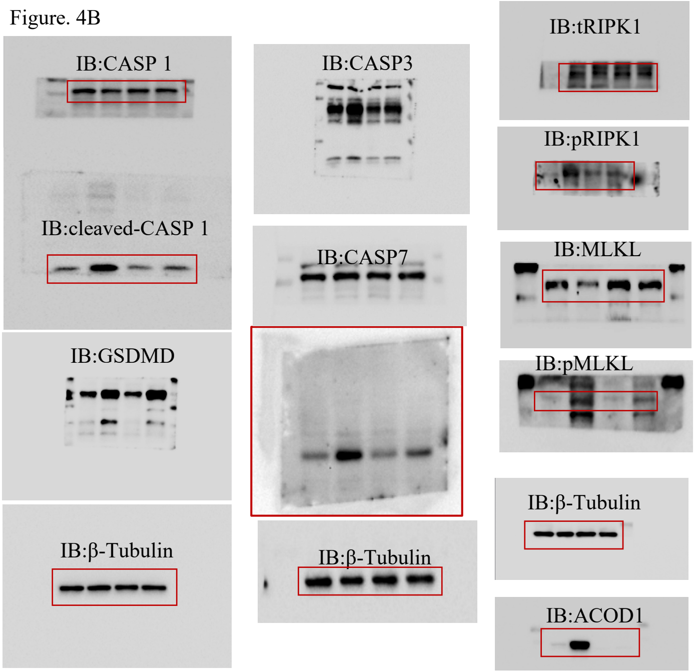

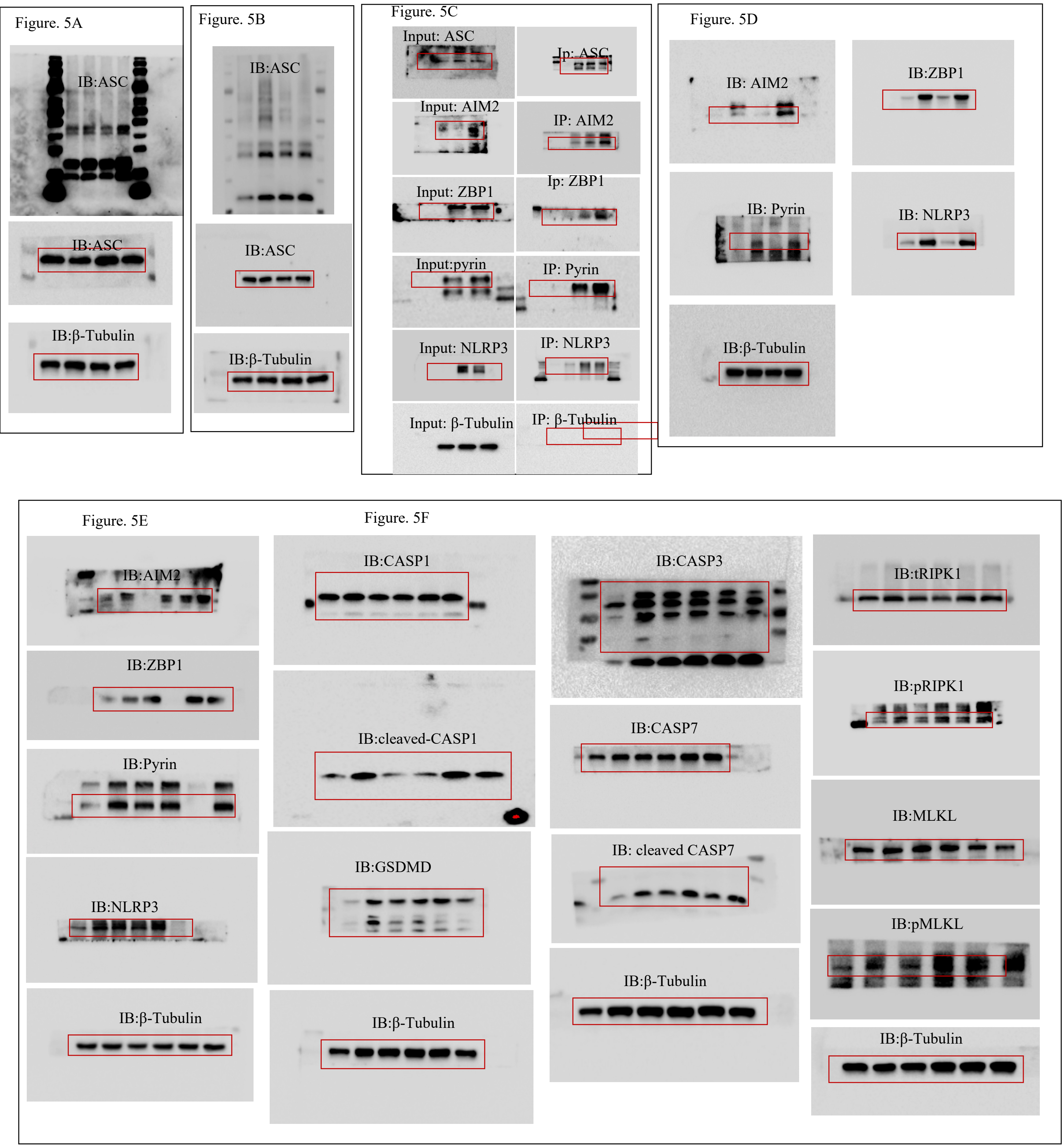
Figure. 5A
IB:ASC
IB:ASC
IB:β-Tubulin
Figure. 5B
IB:ASC
IB:ASC
IB:β-Tubulin
Figure. 5C
Input: ASC
Ip: ASC
Input: AIM2
IP: AIM2
Input: ZBP1
Ip: ZBP1
Input:pyrin
IP: Pyrin
Input: NLRP3
IP: NLRP3
Input: β-Tubulin
IP: β-Tubulin
Figure. 5D
IB: AIM2
IB:ZBP1
IB: Pyrin
IB: NLRP3
IB:β-Tubulin
Figure. 5E
IB:AIM2
IB:ZBP1
IB:Pyrin
IB:NLRP3
IB:β-Tubulin
Figure. 5F
IB:CASP1
IB:cleaved-CASP1
IB:GSDMD
IB:β-Tubulin
IB:CASP3
IB:CASP7
IB: cleaved CASP7
IB:β-Tubulin
IB:tRIPK1
IB:pRIPK1
IB:MLKL
IB:pMLKL
IB:β-Tubulin

Figure. 6B

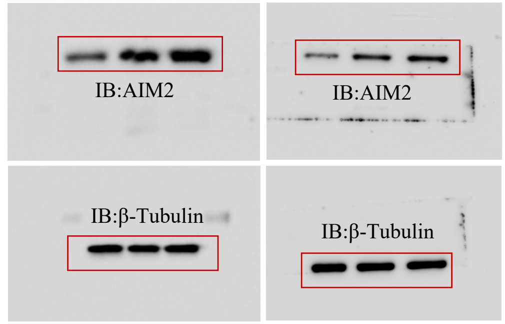

Figure. 6C

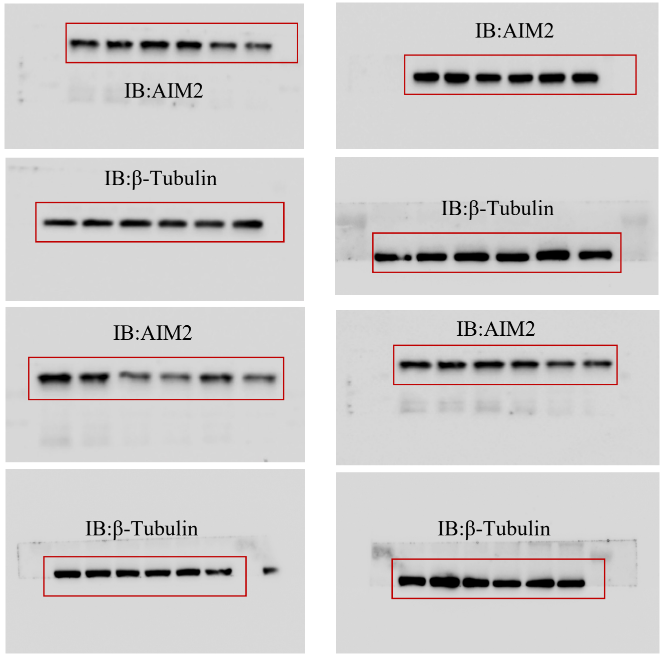

Figure. 6D

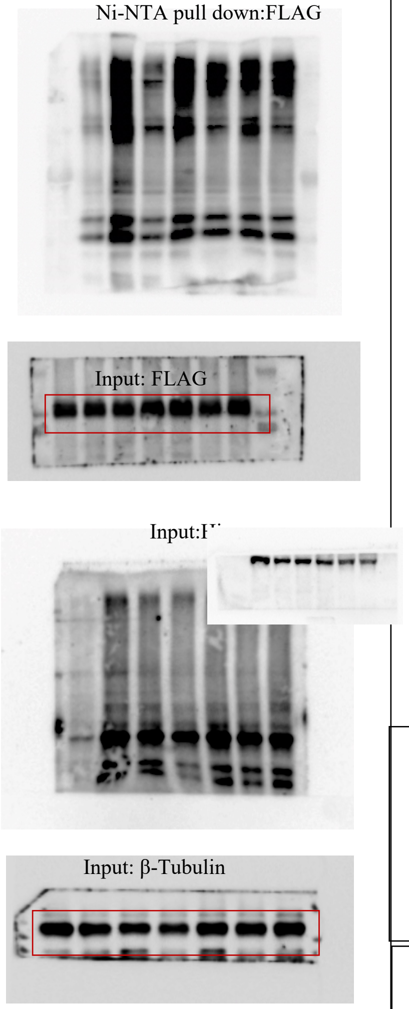

Figure. 6G

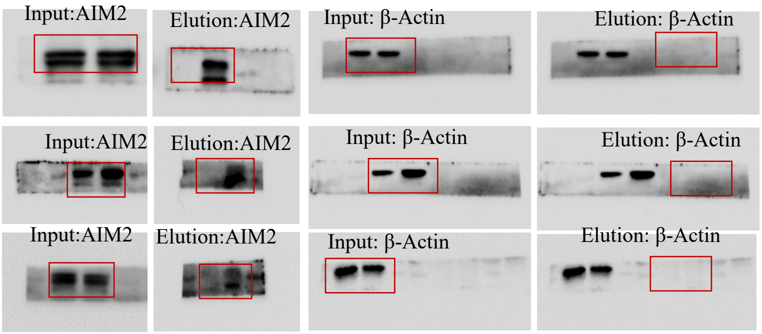

Figure. 6H

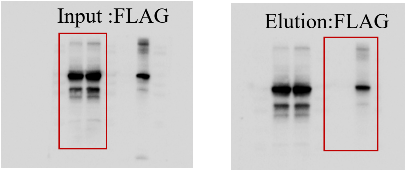

Figure. 6K

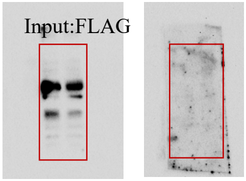

Figure. 5L

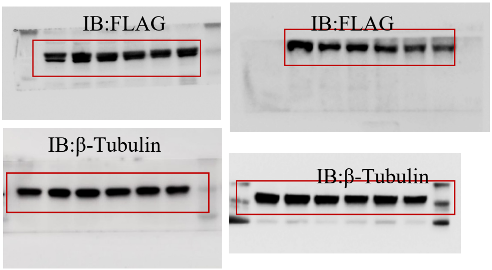

Figure. 7A

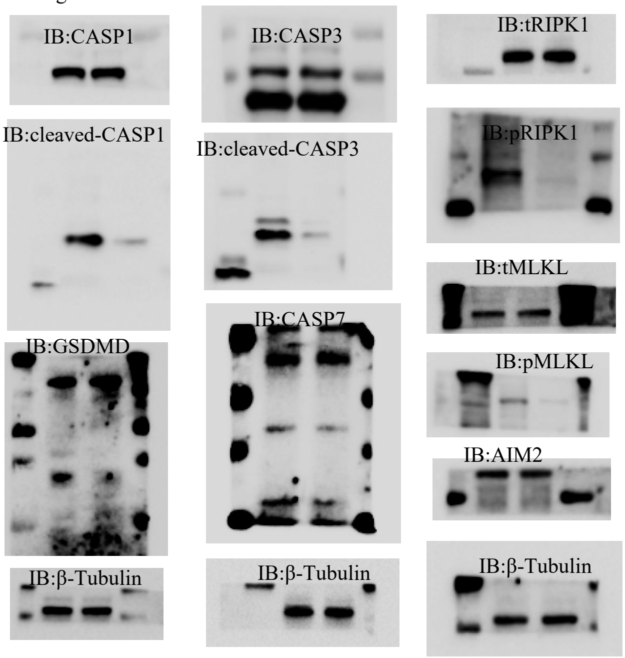

Figure. S7G

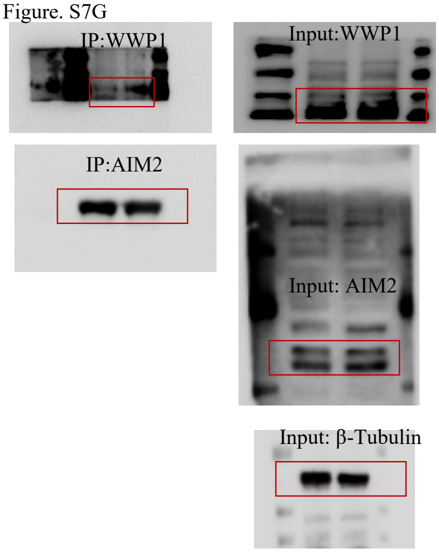

Figure. S2G

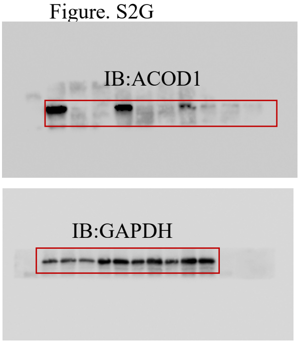

Figure. S4C

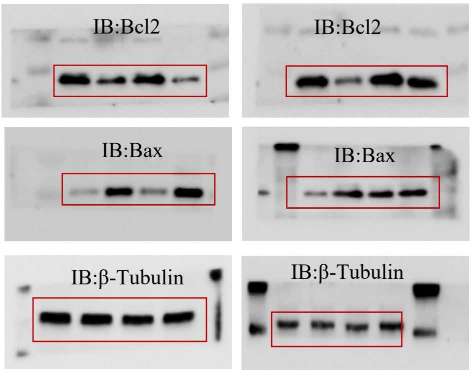

Figure. S7C

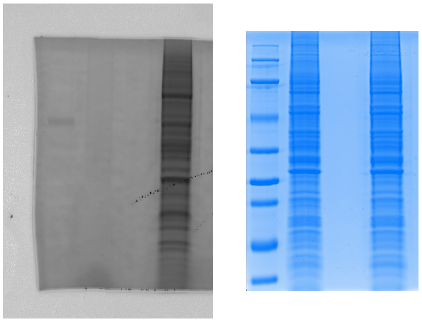

Figure 2F

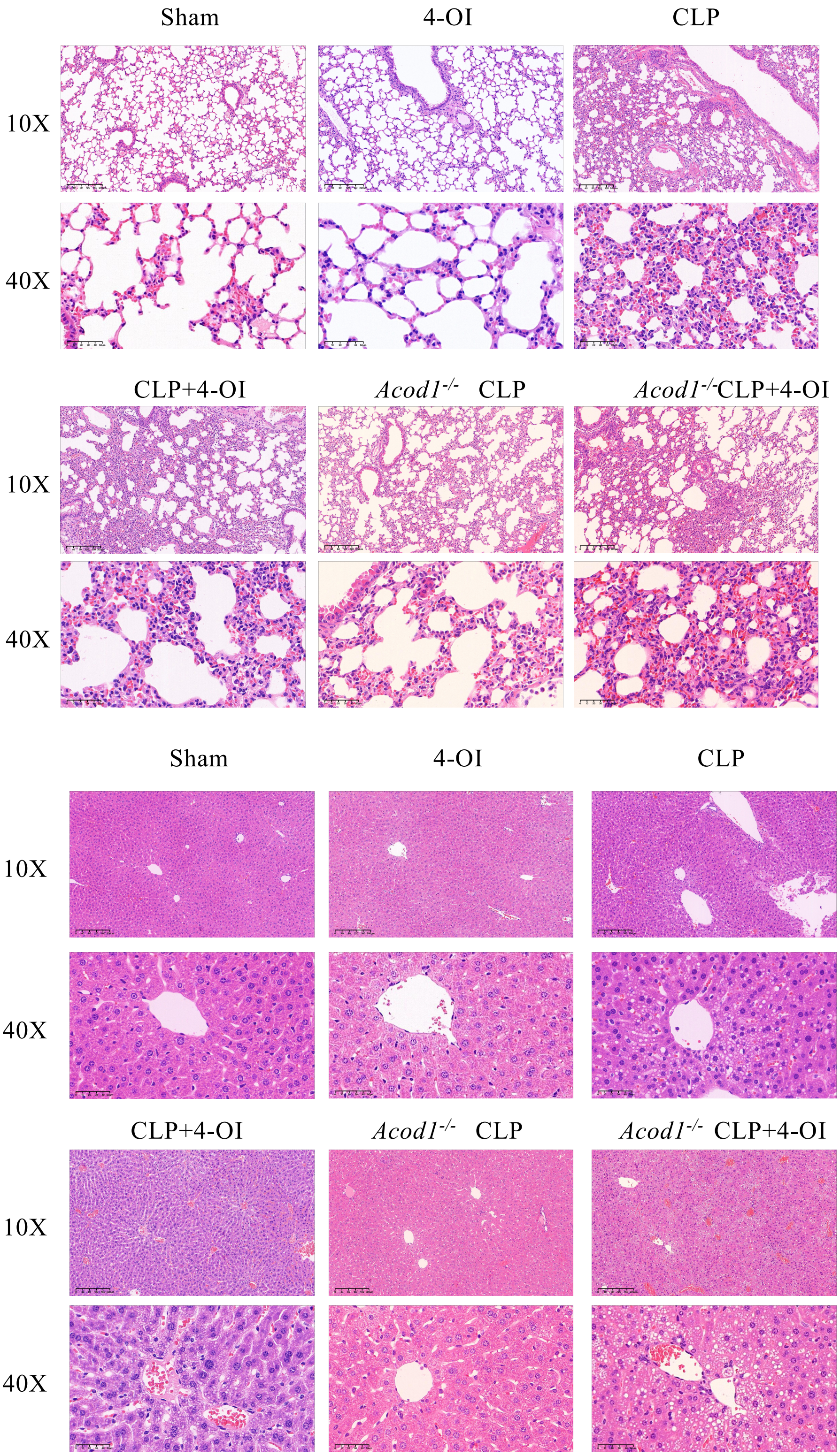
Sham
4-OI
CLP
10X
40X
CLP+4-OI
Acod1-/- CLP
Acod1-/-CLP+4-OI
10X
40X
Sham
4-OI
CLP
10X
40X
CLP+4-OI
Acod1-/- CLP
Acod1-/- CLP+4-OI
10X
40X

Figure 2F

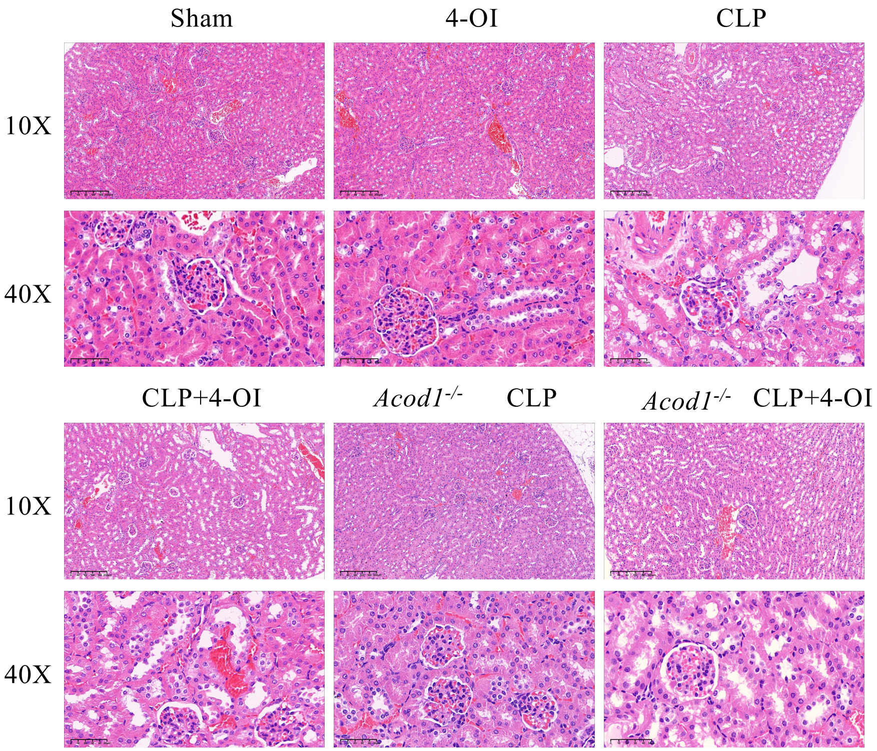

Figure 3F

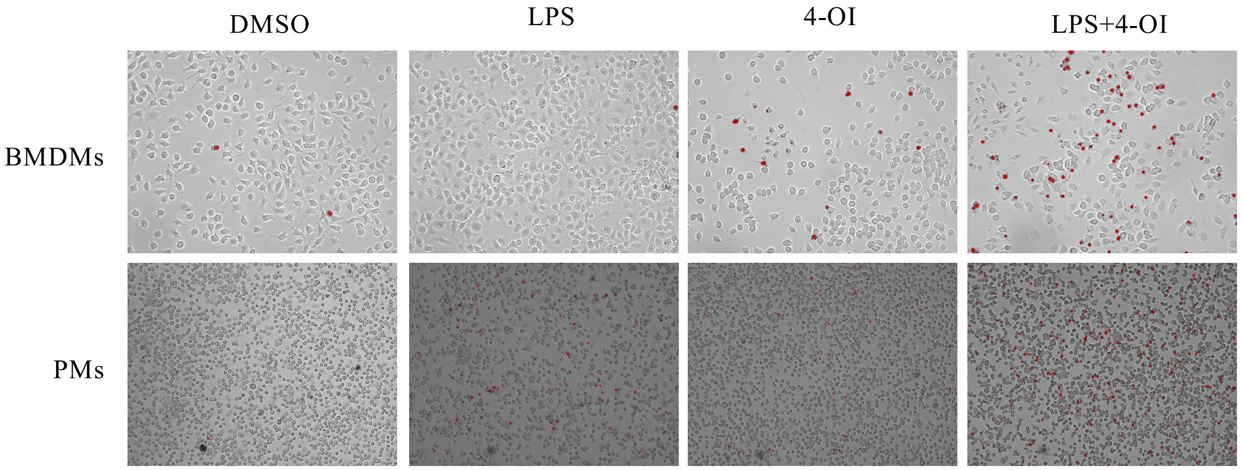

Figure 3I

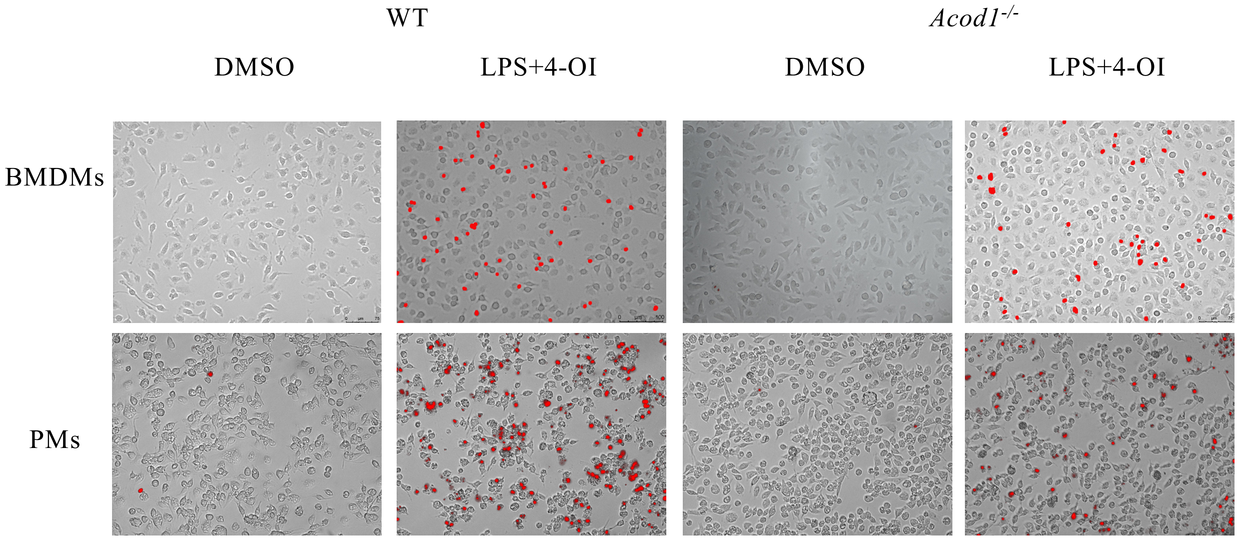

Figure 3O

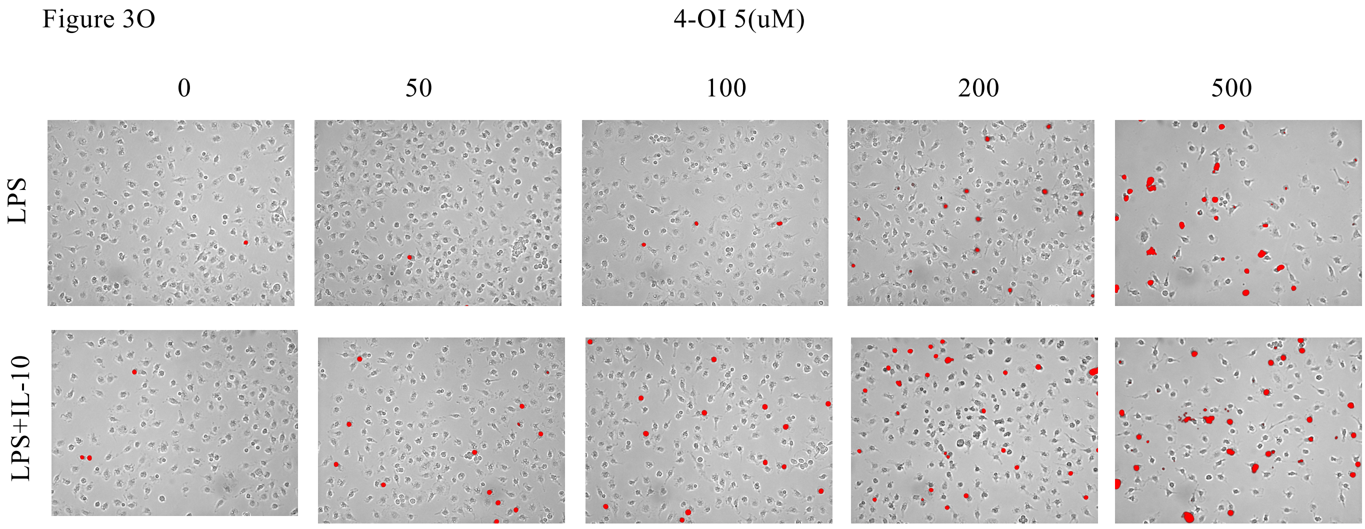

Figure 4C

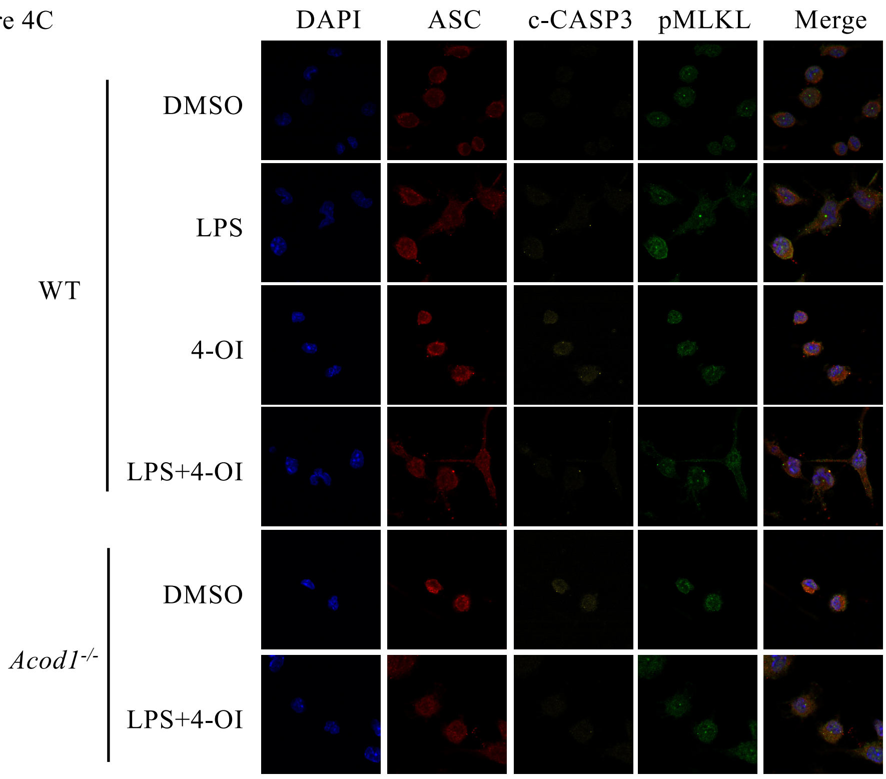

Figure 4E

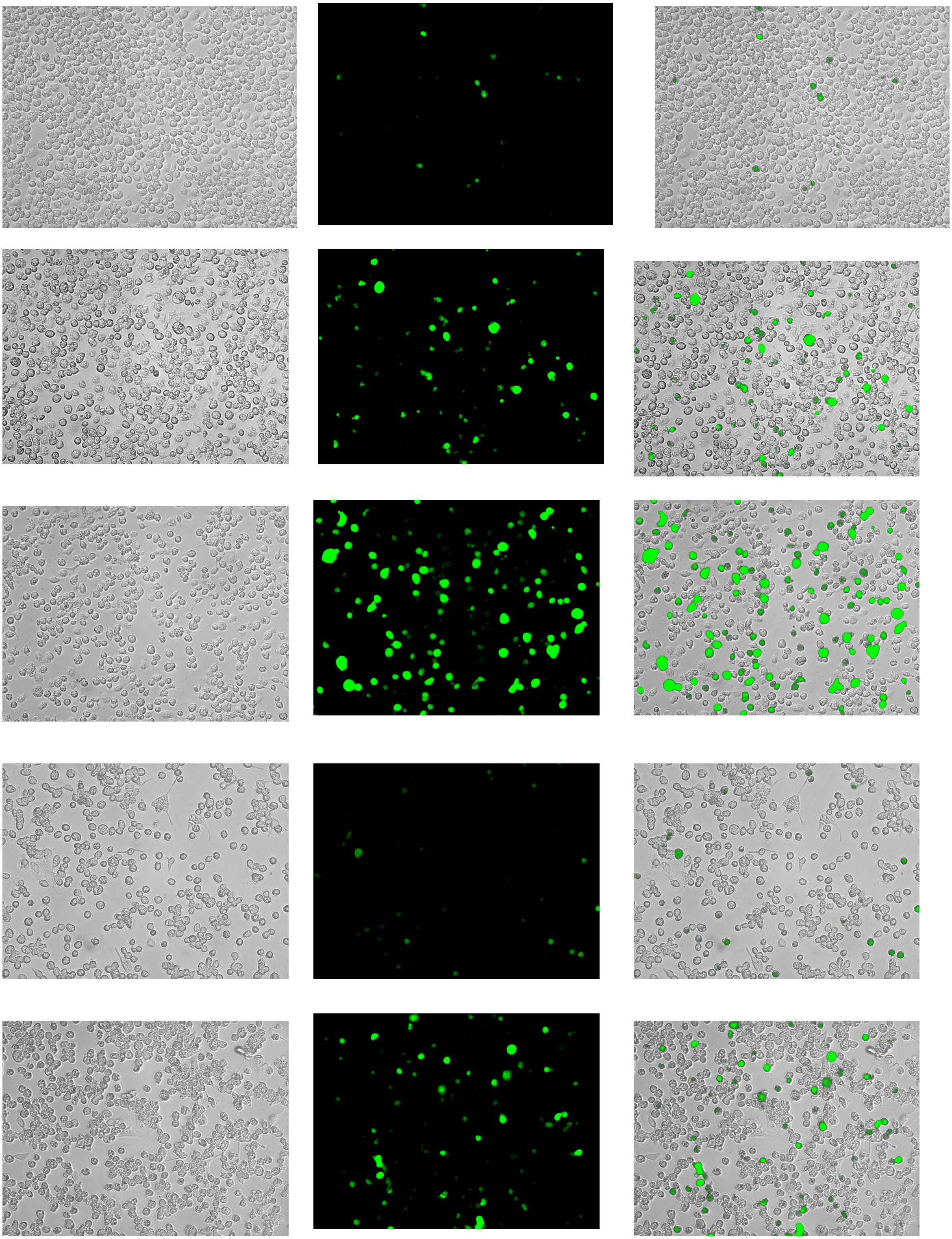

Figre 4E

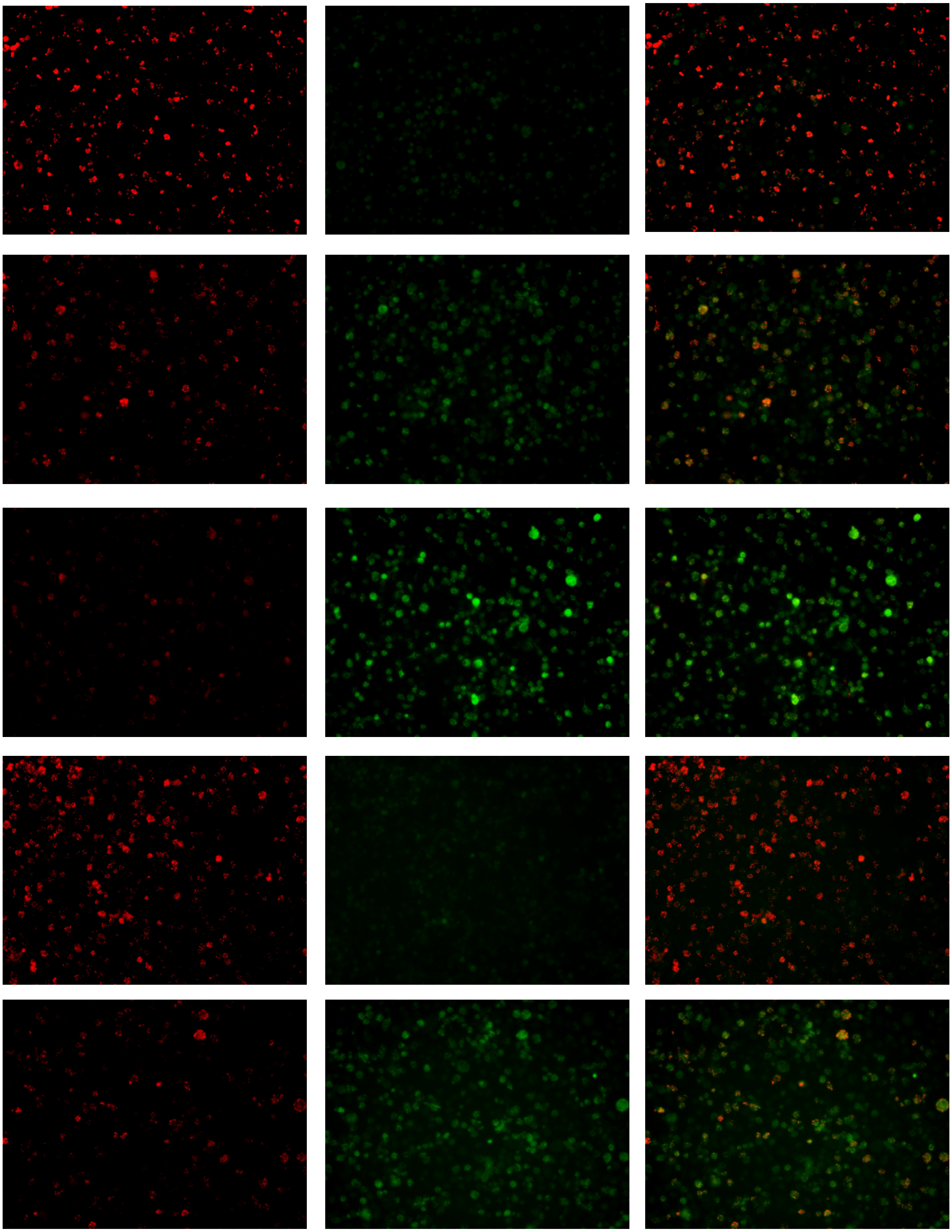

Figure 4I, L

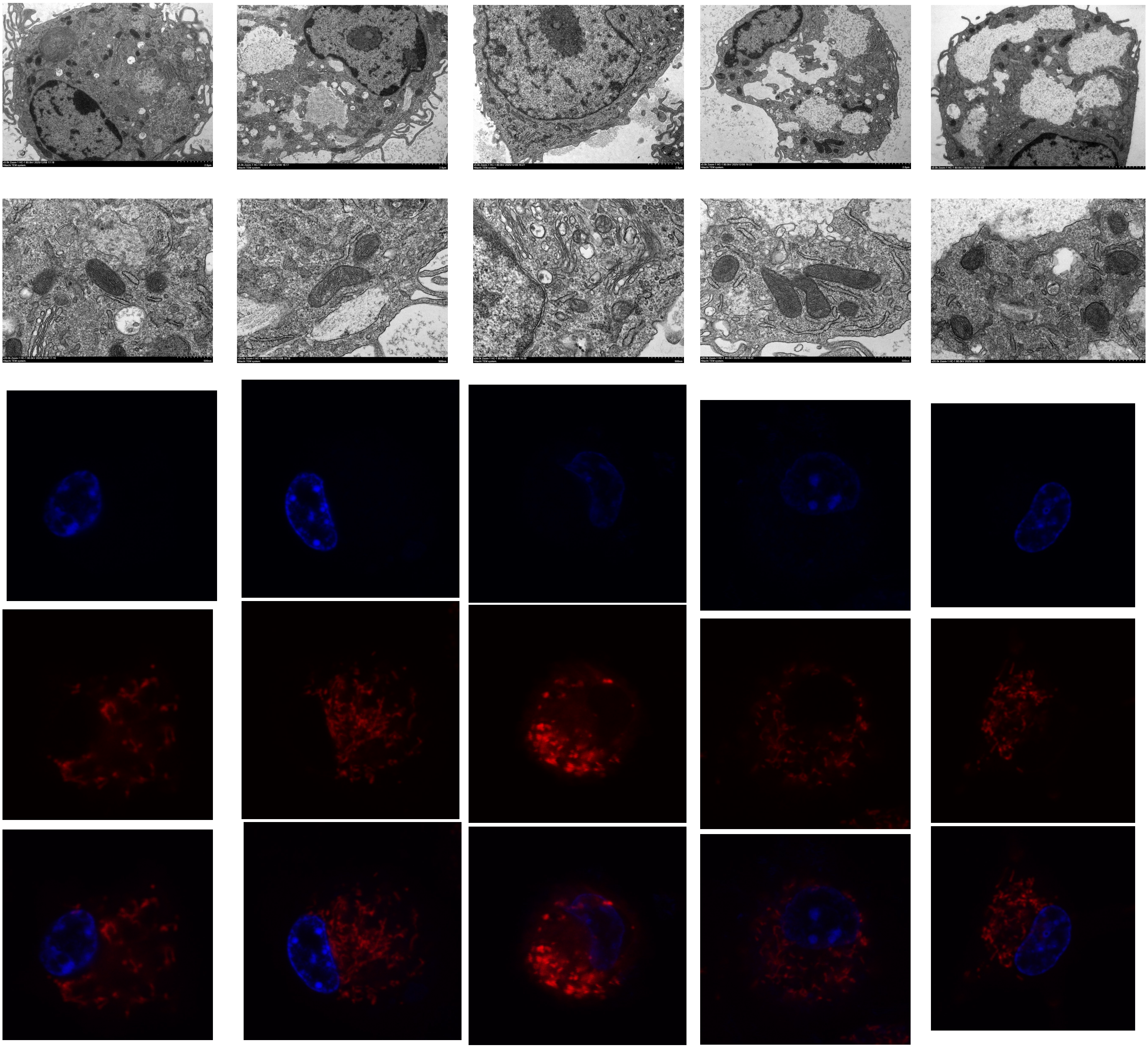

Figure 5H

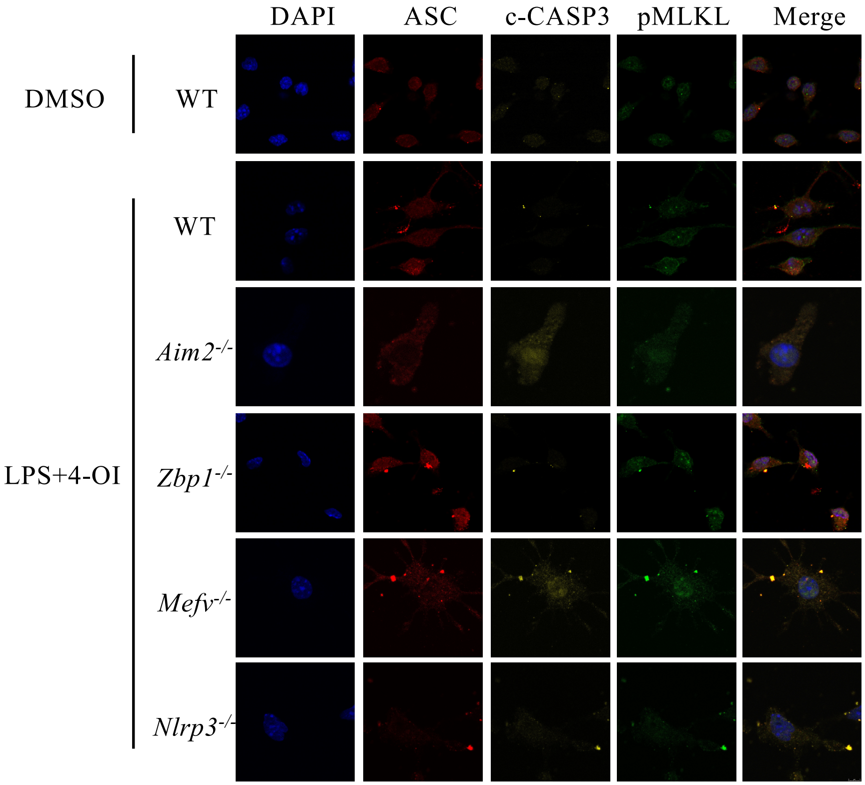
DAPI
ASC
c-CASP3
pMLKL
Merge
DMSO
WT
LPS+4-OI
WT
Aim2-/-
Zbp1-/-
Mefv-/-
Nlrp3-/-

Figure 5K

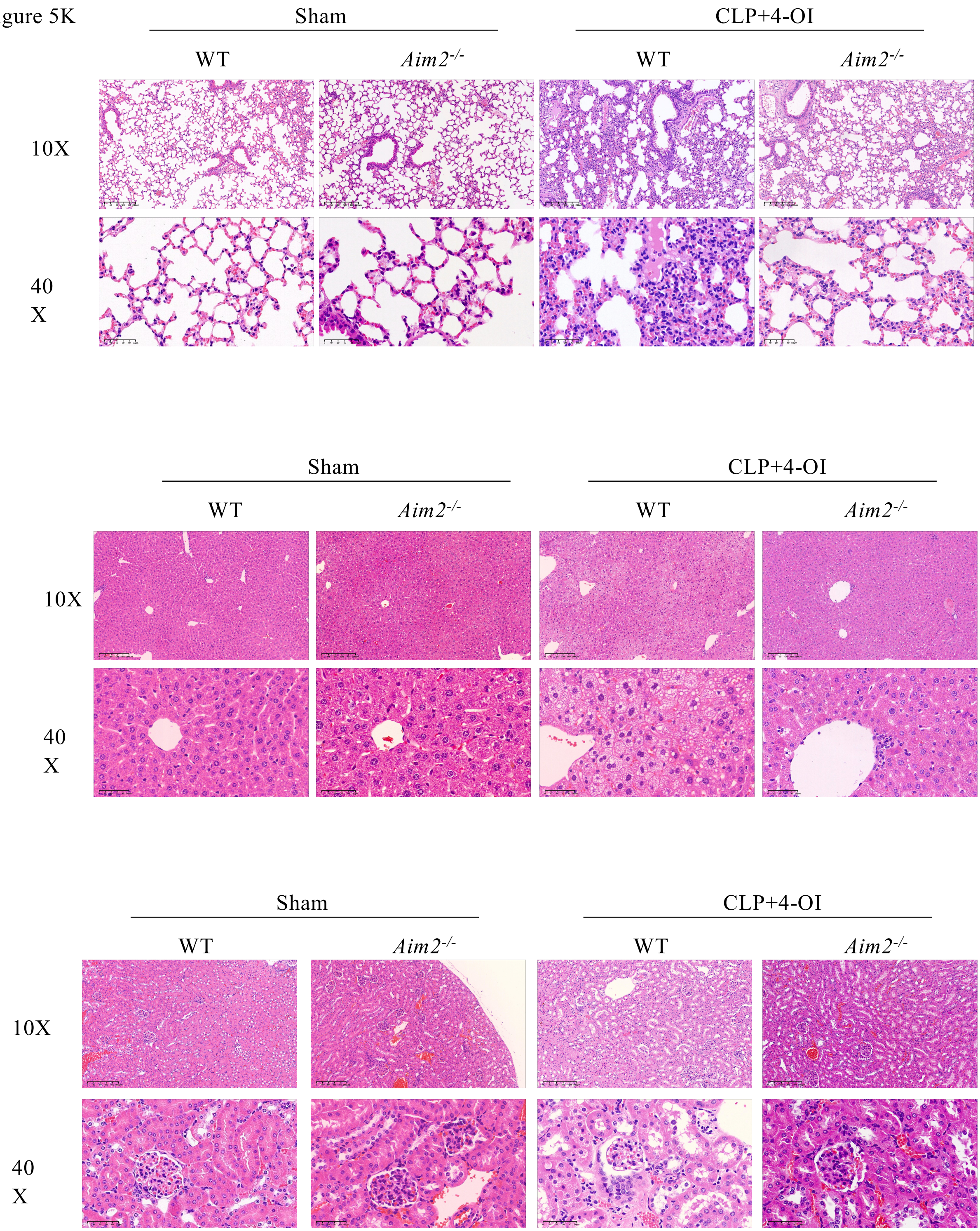
Sham
CLP+4-OI
WT
Aim2-/-
WT
Aim2-/-
10X
40X
Sham
CLP+4-OI
WT
Aim2-/-
WT
Aim2-/-
10X
40X
Sham
CLP+4-OI
WT
Aim2-/-
WT
Aim2-/-
10X
40X

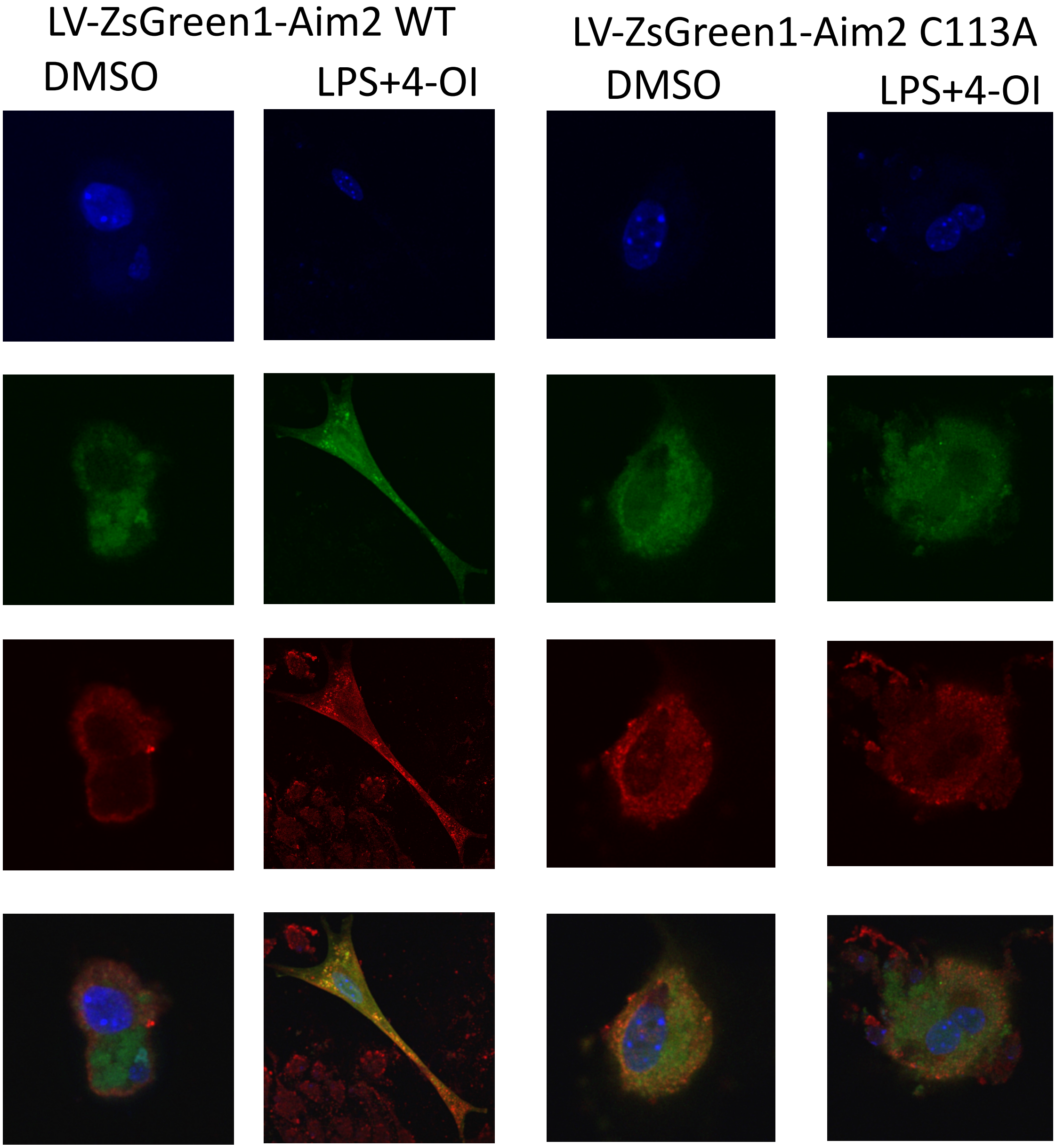
LV-ZsGreen1-Aim2 WT
DMSO
LPS+4-OI
LV-ZsGreen1-Aim2 C113A
DMSO
LPS+4-OI

Figure S1B

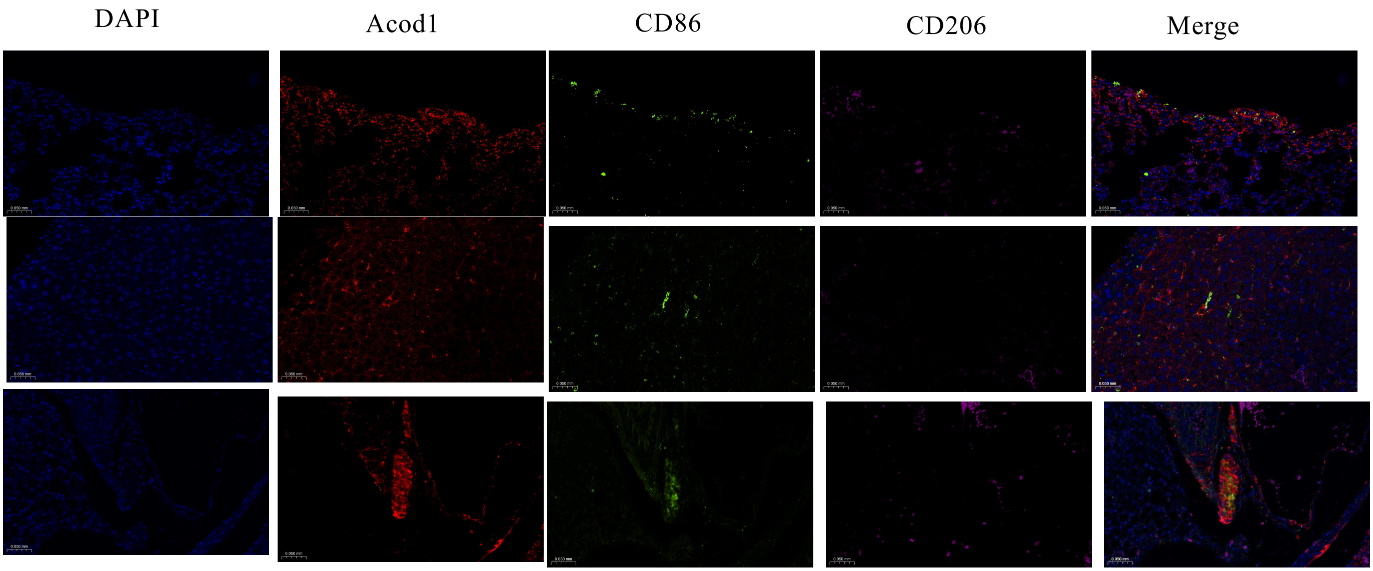

Figure S3M

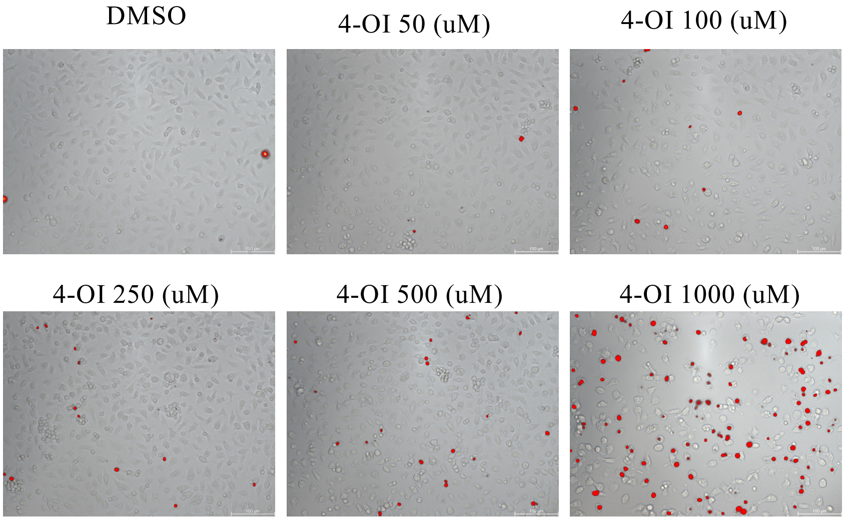

Figure S3O

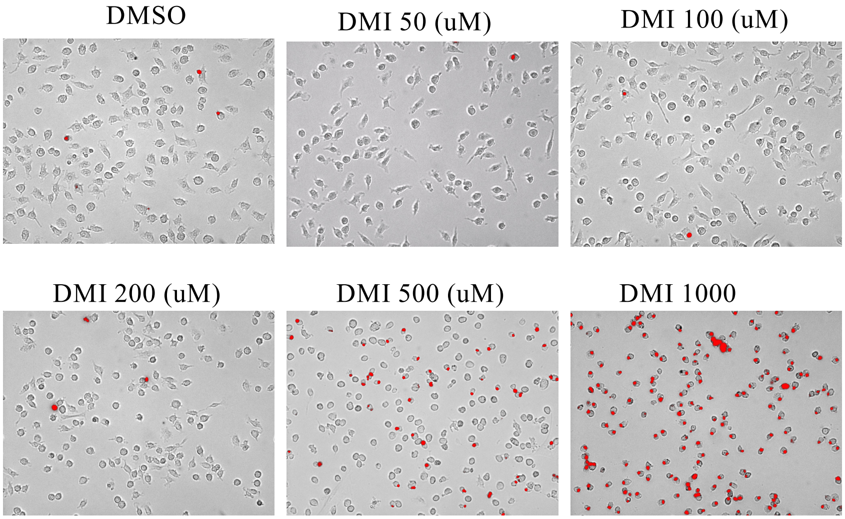

Figure S3Q

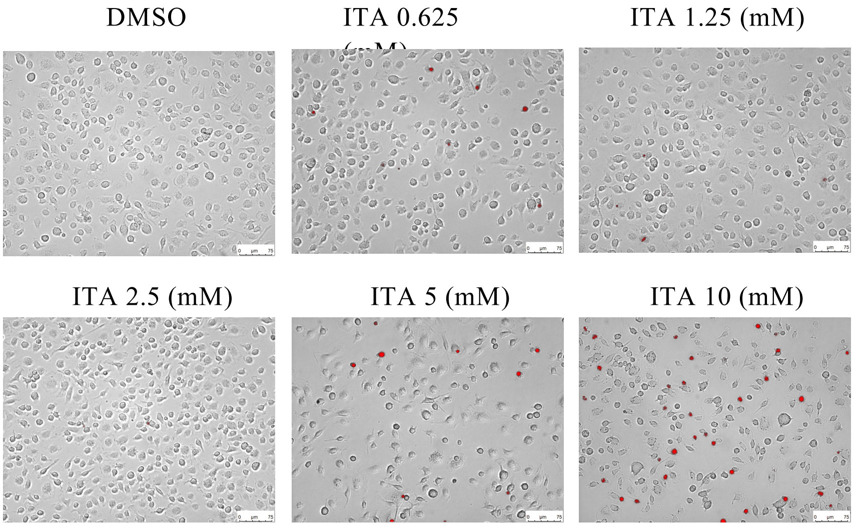

Figure S55E

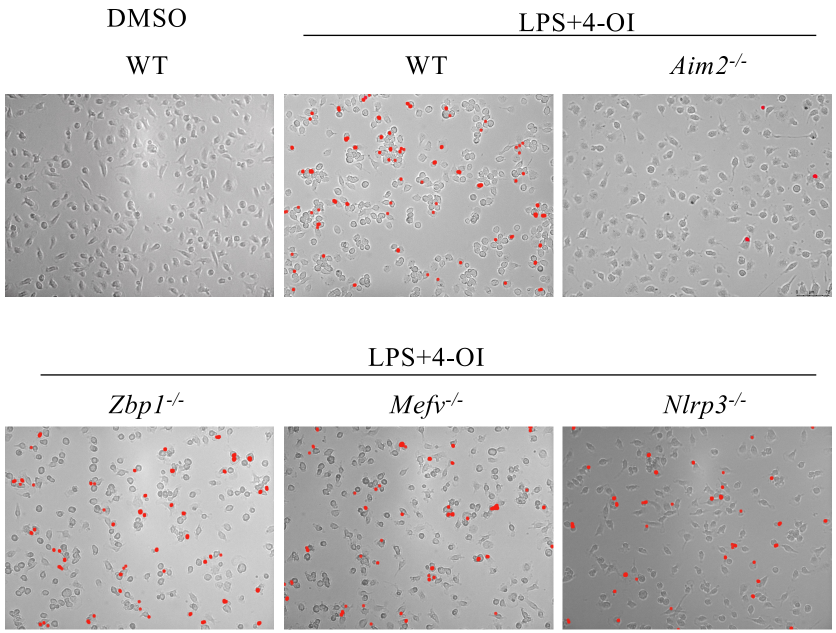

Figure S5J

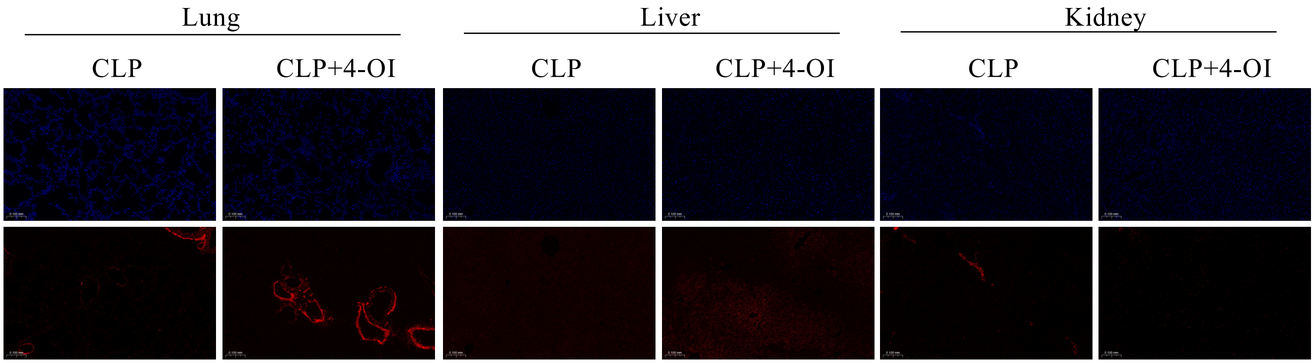

Figure S6A

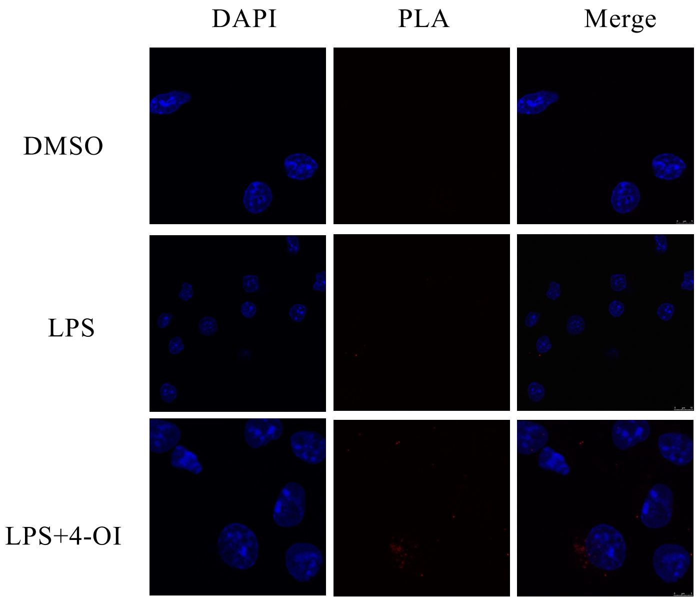

Figure S6B

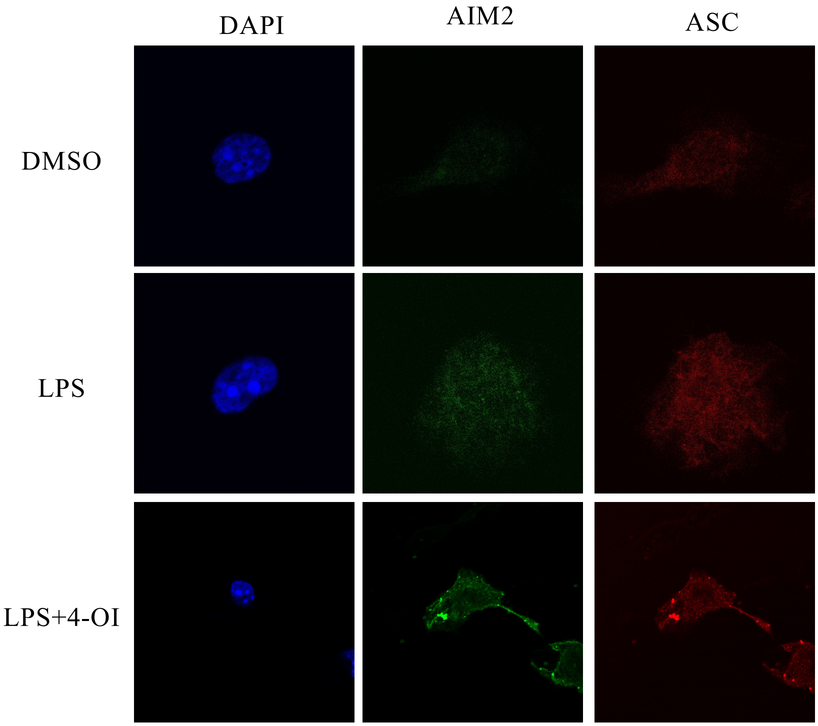

Figure S6A

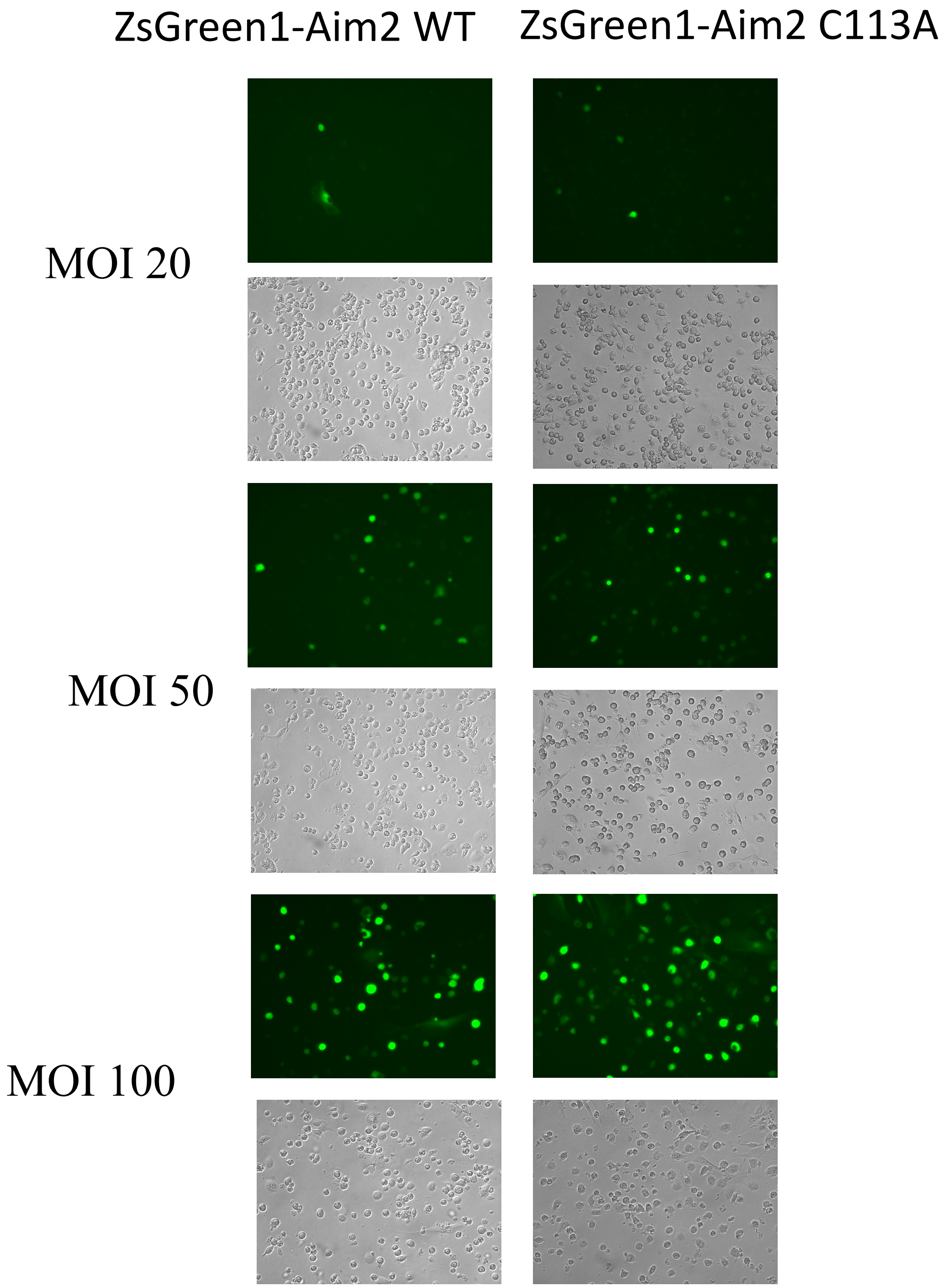

Supplement: Supplementary file 2 — original images [file 41423_2026_1414_MOESM2_ESM.pdf]
