# Supplementary material for: The alkylation of AIM2 by itaconate mediates macrophage PANoptosis during sepsis
Source: Cell Mol Immunol. 2026 May 12;23(6):619–34. doi: 10.1038/s41423-026-01414-x (PMC13222358; doi:10.1038/s41423-026-01414-x)
Supplement: Supplementary file 3 — Supplementary tables [file 41423_2026_1414_MOESM3_ESM.docx]

**Supplementary tables**

**Supplementary Table 1. Clinical characteristics of healthy patients and sepsis**

| Index | Control group (n=7) | Sepsis group (n=7) | *P* value |
| --- | --- | --- | --- |
| Age (years) | 59.57±6.26 | 63.71±13.57 | 0.618 |
| Sex (male/female) | 3/4 | 5/2 | 0.515 |
| APACHE II | N/A | 20.29±6.10 | N/A |
| SOFA | N/A | 12.4±5.19 | N/A |
| GCS | N/A | 10.14±3.13 | N/A |

Notes: Between-group comparison: *P*-value reported based on independent sample t-test and Wilcoxon rank sum test for quantitative variables and chi-square test for qualitative variables. Data are shown as mean ± standard deviation.
Abbreviations: APACHE II, Acute Physiology and Chronic Health Evaluation; SOFA, Sequential Organ Failure Assessment; GCS, Glasgow Coma Scale.

**Supplementary Table 2. The data from GSE95233**

| Index | | Survivor (n = 34) | Non-survival (n = 17) | *P* value |
| --- | --- | --- | --- | --- |
| Age (years) | | 62.85 ± 16.21 | 60.88 ± 12.82 | 0.664 |
| Sex (male/female/half) | | 25/9/0 | 6/9/2 | 0.011 |
| *ACDO1*  expression | D1 | 3.39 ± 0.24 | 3.46 ± 0.34 | 0.3676 |
|  | D2 | 3.42 ± 0.16 | 3.45 ± 0.20 | 0.7358 |
|  | D3 | 3.26 ± 0.20 | 3.57 ± 0.36 | 0.0045 |

Notes: Between group comparison: *P*-value reported based on independent sample t-test and Wilcoxon rank sum test for quantitative variables and chi-square test for qualitative variables. Data are shown as mean ± standard deviation.

Abbreviations: D1, day 1 post-admission; D2, day 2 post-admission; D3, day 3 post-admission.

**Supplementary Table 3. The data from GSE236713**

| Index | | Survivor (n = 118) | Non-survival (n = 46) | *P* value |
| --- | --- | --- | --- | --- |
| Age (years) | | Not mentioned | Not mentioned | N/A |
| Sex (male/female) | | 68/50 | 28/18 | 0.705 |
| *ACDO1*  expression | D1 | 0.50 ± 1.36 | 0.29 ± 1.36 | 0.3784 |
|  | D2 | 0.20 ± 1.32 | 0.37 ± 1.30 | 0.5387 |
|  | D5 | -0.00 ± 1.356 | 0.20 ± 1.60 | 0.6113 |

Notes: Between group comparison: *P*-value reported based on independent sample t-test and Wilcoxon rank sum test for quantitative variables and chi-square test for qualitative variables. Data are shown as mean ± standard deviation.

Abbreviations: D1, day 1 post-admission to ICU; D2, day 2 post-admission to ICU; D5, day 5 post-admission to ICU.

**Supplementary Table 4. Antibody-Reagent-Software**

| REAGENT or RESOURCE | SOURCE | IDENTIFIER |
| --- | --- | --- |
| Antibodies |  |  |
| Anti-GAPDH | Proteintech | Cat# 60004-1-Ig; RRID: AB_2107436 |
| Anti-β-tubulin | Absin | Cat #abs830032 |
| Anti-ACOD1 | Cell Signaling Technology | Cat# 77510S; RRID: AB_2799901 |
| Anti-MLKL | Cell Signaling Technology | Cat# 37705S; RRID: AB_2799118 |
| Anti-p-MLKL | Cell Signaling Technology | Cat# 37333S; RRID: AB_2799112 |
| Anti-RIPK1 | Cell Signaling Technology | Cat# 3493S; RRID: AB_2305314 |
| Anti-p-RIPK1 | Affinity | Cat# AF2398; RRID: AB_2845412 |
| Anti-Caspase-1 | Cell Signaling Technology | Cat# 24232S; RRID: AB_2890194 |
| Anti- cleaved-Caspase-1 | Cell Signaling Technology | Cat# 89332S; RRID: AB_2923067 |
| Anti-Caspase-3 | Cell Signaling Technology | Cat# 14220S; RRID: AB_2798429 |
| Anti-Caspase-3 p17 | Santa Cruz Biotechnology | Cat# 373730; RRID: AB_10918110 |
| Anti-Caspase-7 | Cell Signaling Technology | Cat# 12827S; RRID: AB_2687912 |
| Anti-cleaved-Caspase-7 | Cell Signaling Technology | Cat# 9491S; RRID: AB_2068144 |
| Anti-GSDMD | Cell Signaling Technology | Cat# 39754T; RRID: AB_2916333 |
| Anti-cleaved-GSDMD | Cell Signaling Technology | Cat# 10137; RRID: AB_2923068 |
| Anti-AIM2 | Cell Signaling Technology | Cat# 53491S; RRID: AB_3065230 |
| Anti-AIM2 | Proteintech | Cat# 20590-1-AP; RRID: AB_10694420) |
| Anti-Pyrin | Abcam | Cat# ab195975; RRID: AB_3674164 |
| Anti-ZBP1 | Adipogen | Cat# AG-20B-0010; RRID: AB_2490191 |
| Anti-NLRP3 | Proteintech | Cat# 68102-1-Ig; RRID: AB_2923634 |
| Anti-ASC | Cell Signaling Technology | Cat# 67824; RRID: AB_2799736 |
| Anti-ASC | Abcam | Cat# ab175449; RRID: AB_3096354 |
| Anti-BAX | Abmart | Cat# T40051; RRID: AB_2910262 |
| Anti-BCL2 | Abmart | Cat# T40056; RRID: AB_2929011 |
| Anti-WWP1 | Affinity | Cat#DF12506; RRID: AB_2845311 |
| Anti-β-actin | Cell Signaling Technology | Cat# 93473; RRID: AB_3099713 |
| Anti-Flag | Abcam | Cat# ab205606; RRID: AB_2916341 |
| Anti-His | Cwbio | Cat# CW0286M |
| Chemicals, Peptides, and Recombinant Proteins | | |
| 4-Octyl Itaconate | MedChemExpress | Cat# HY-112675 |
| Itaconic acid | Sigma-Aldrich | Cat# I29204 |
| Dimethyl itaconate | Sigma-Aldrich | Cat#5 92498 |
| Itaconate-alkyne (ITalk) | ChomiX Biotech Co.,Ltd | Cat# CMX210035 |
| LPS | Sigma-Aldrich | Cat# L2630 |
| Sytox | Invitrogen | Cat# S7020 |
| ROS | Beyotime | Cat# S0033S |
| JC-1 | Beyotime | Cat# C2006 |
| Mito-tracker Red | Beyotime | Cat# C1035 |
| IL-10 | MedChemExpress | Cat# HY-149394 |
| BeyoMag™ Anti-His Magnetic Beads | Beyotime | Cat# P2135 |
| Cycloheximide (CHX) | MedChemExpress | Cat# HY-12320 |
| EZ Cell Transfection Reagent | Shanghai Life-ilab Biotech Co.,Ltd | Cat# AC04L091 |
| Halt Protease Inhibitor Cocktail (100X) | Thermo Fisher Scientific | Cat# 78430 |
| TBTA ligand | Strem | Cat# 07-3215 |
| Biotin-Azide | ChemPep | Cat# 271604 |
| Tris (2-carboxyethyl) phosphine hydrochloride (TCEP) | Sigma Aldrich | Cat# 4706 |
| Streptavidin-agarose beads | Thermo Fisher Scientific | Cat# 20353 |
| EGTA | Sigma Aldrich | Cat# E3889 |
| EDTA | Thermo Fisher Scientific | Cat# C11843 |
| CHAPS | Sigma Aldrich | Cat# V900480 |
| PMSF | Sigma Aldrich | Cat# P7626 |
| 20mM HEPES-KOH | Shanghai yuanye Bio-Technology Co., Ltd | Cat# R27420 |
| Mouse M-CSF Recombinant Protein | PeproTech | Cat# 315-02 |
| DMEM, high glucose | Gibco | Cat# C11995500BT |
| RPMI 1640 | Gibco | Cat# C11875500BT |
| TRIzol | Thermo Fisher Scientific | Cat# 15596018 |
| Critical commercial assays |  |  |
| BCA Protein Assay Kit | NCM Biotech | Cat# WB6501 |
| Mouse IL-1β ELISA kit | ThermoFisher | Cat# 88–7013 |
| Mouse IL-6 ELISA kit | ThermoFisher | Cat# 88-7064 |
| Mouse TNF-α ELISA kit | ThermoFisher | Cat# 88-7324 |
| Mouse AIM2 ELISA kit | YOBIBIO | Cat# U96-1618E |
| Malondialdehyde (MDA) assay kit | Nanjing Jiancheng Bioengineering Institute | Cat# A003-1-2 |
| Aspartate aminotransferase (AST) Assay Kit | Nanjing Jiancheng Bioengineering Institute | Cat# C010-2-1 |
| Alanine aminotransferase (ALT) Assay Kit | Nanjing Jiancheng Bioengineering Institute | Cat# C009-2-1 |
| Blood urea nitrogen (BUN) assay kit | Nanjing Jiancheng Bioengineering Institute | Cat# C013-2-1 |
| Creatinine (Cr) Assay kit | Nanjing Jiancheng Bioengineering Institute | Cat# C011-2-1 |
| Cell Counting Kit-8 | DOJINDO | Cat# KR675 |
| Experimental models: Cell lines |  |  |
| HEK293T | ATCC | Cat# CRL-11268; RRID: CVCL_1926 |
| Mouse: RAW264.7 | From Li Jinbao's lab | N/A |
| Experimental models: Organisms/strains |  |  |
| Mouse: C57BL/6J | Shanghai Legen Biotechnology Co., Ltd | N/A |
| Mouse: *Acod11^-/-^* C57BL/6J | Cyagen Biosciences Inc | N/A |
| Mouse: *Aim2^-/-^* C57BL/6J | Cyagen Biosciences Inc | N/A |
| Mouse: *Zbp1^-/-^* C57BL/6J | Cyagen Biosciences Inc | N/A |
| Mouse: *Mefv^-/-^* C57BL/6J | Cyagen Biosciences Inc | N/A |
| Mouse: *Nlrp3^-/-^* C57BL/6J | Cyagen Biosciences Inc | N/A |
| Oligonucleotides |  |  |
| Primers for qPCR analysis, see Table 4 | This paper | N/A |
| Recombinant DNA |  |  |
| pCMV-AIM2-FLAG | This paper | N/A |
| pCMV-AIM2 C113- FLAG | This paper | N/A |
| LV-ZsGreen1-Aim2 WT | This paper | N/A |
| LV-ZsGreen1-Aim2 C113A | This paper | N/A |
| Software and algorithms | | |
| GraphPad Prism v8.4.3 | GraphPad | https://www.graphpad.com/ |
| ImageJ | National Institutes of Health | https://imagej.nih.gov/ij/ |
| Image Lab (Version 6.0.1) | Bio-Rad | https://www.bio-rad.com/ |
| AutoDock Tools 1.5.2 | National Institutes of Health | https://ccsb.scripps.edu/ |

**Supplementary Table 5. Primers used for qRT-PCR analysis**

| Primers | Sequence |
| --- | --- |
| 18S-mouse-F | 5’- TTCCGATAACGAACGAGACTCT - 3’ |
| 18S-mouse-R | 5’- TGGCTGAACGCCACTTGTC - 3’ |
| *IL-1β*-mouse-F | 5’- GCAACTGTTCCTGAACTCAACT- 3’ |
| *IL-1β*-mouse-R | 5’- ATCTTTTGGGGTCCGTCAACT- 3’ |
| *TNF-α*-mouse-F | 5’- CCCTCACACTCAGATCATCTTCT- 3’ |
| *TNF-α*-mouse-R | 5’- GCTACGACGTGGGCTACAG- 3’ |
| *IL-6*-mouse-F | 5’- TAGTCCTTCCTACCCCAATTTCC- 3’ |
| *IL-6*-mouse-R | 5’- TTGGTCCTTAGCCACTCCTTC- 3’ |
| *Acod1*-mouse-F | 5’- TGCTGCGTGCGTCCAAGTTT - 3 |
| *Acod1*-mouse-R | 5’- GGGGCTTAGTCTGAGTGGC - 3 |
| *Aim2*-mouse-F | 5’-TAACTTCATGGAGGTCACCAGTTC- 3’ |
| *Aim2*-mouse-R | 5’- GGAGTTTCCCTGGCTCTCTTAAC- 3’ |
| *Zbp1*-mouse-F | 5’-AAGAGTCCCCTGCGATTATTTG - 3’ |
| *Zbp1*-mouse-R | 5’-TCTGGATGGCGTTTGAATTGG - 3’ |
| *Mefv*-mouse-F | 5’-CGACCCAGGAGTCTTGAAATTA- 3’ |
| *Mefv* -mouse-R | 5’-GTCCTTCCATTCAGAGTAGCTGTT- 3’ |
| *Nlrp3*-mouse-F | 5’-ATTACCCGCCCGAGAAAGG - 3’ |
| *Nlrp3*-mouse-R | 5’-TCGCAGCAAAGATCCACACAG - 3’ |
